# Effects of epigenetic age acceleration on kidney function: a Mendelian randomization study

**DOI:** 10.1186/s13148-023-01476-y

**Published:** 2023-04-08

**Authors:** Yang Pan, Xiao Sun, Zhijie Huang, Ruiyuan Zhang, Changwei Li, Amanda H. Anderson, James P. Lash, Tanika N. Kelly

**Affiliations:** 1 Division of Nephrology, Department of Medicine, College of Medicine, University of Illinois at Chicago, 820 S Wood Street, Chicago, IL 60607 USA; 2 Department of Epidemiology, School of Public Health and Tropical Medicine, Tulane University, New Orleans, LA USA

**Keywords:** Epigenetic age acceleration, Kidney function, Mendelian randomization, eGFR, CKD

## Abstract

**Background:**

Previous studies have reported cross-sectional associations between measures of epigenetic age acceleration (EAA) and kidney function phenotypes. However, the temporal and potentially causal relationships between these variables remain unclear. We conducted a bidirectional two-sample Mendelian randomization study of EAA and kidney function. Genetic instruments for EAA and estimate glomerular filtration rate (eGFR) were identified from previous genome-wide association study (GWAS) meta-analyses of European-ancestry participants. Causal effects of EAA on kidney function and kidney function on EAA were assessed through summary-based Mendelian randomization utilizing data from the CKDGen GWAS meta-analysis of log-transformed estimated glomerular filtration rate (log-eGFR; *n* = 5,67,460) and GWAS meta-analyses of EAA (*n* = 34,710). An allele score-based Mendelian randomization leveraging individual-level data from UK Biobank participants (*n* = 4,33,462) further examined the effects of EAA on kidney function.

**Results:**

Using summary-based Mendelian randomization, we found that each 5 year increase in intrinsic EAA (IEAA) and GrimAge acceleration (GrimAA) was associated with − 0.01 and − 0.02 unit decreases in log-eGFR, respectively (*P* = 0.02 and *P* = 0.09, respectively), findings which were strongly supported by allele-based Mendelian randomization study (both *P* < 0.001). Summary-based Mendelian randomization identified 24% increased odds of CKD with each 5-unit increase in IEAA (*P* = 0.05), with consistent findings observed in allele score-based analysis (*P* = 0.07). Reverse-direction Mendelian randomization identified potentially causal effects of decreased kidney function on HannumAge acceleration (HannumAA), GrimAA, and PhenoAge acceleration (PhenoAA), conferring 3.14, 1.99, and 2.88 year decreases in HanumAA, GrimAA, and PhenoAA, respectively (*P* = 0.003, 0.05, and 0.002, respectively) with each 1-unit increase in log-eGFR.

**Conclusion:**

This study supports bidirectional causal relationships between EAA and kidney function, pointing to potential prevention and therapeutic strategies.

**Supplementary Information:**

The online version contains supplementary material available at 10.1186/s13148-023-01476-y.

## Background

Chronic kidney disease (CKD) is an important determinant of morbidity and all-cause mortality worldwide [1]. Disproportionately affecting older adults [2], the public health and socioeconomic burdens posed by CKD are expected to grow in tandem with global population aging. Within the older adult population, however, it has been challenging to separate the effects of natural aging processes from various comorbidities on kidney function decline [3]. The recent debate over the use of an age-adapted diagnosis for CKD stresses the clinical relevance of improving our knowledge of the relationship between the biological aging and the kidney [4], which is largely incomplete [5].

In recent years, epigenetic age has become the gold standard measure of biological aging [6, 7]. Based on DNA methylation measured at numerous sites across the human genome, epigenetic clocks have been shown to better predict both chronological age and mortality compared to conventional (e.g., telomere length) and emerging (e.g., omics-based) biomarkers [6]. Epigenetic age acceleration (EAA), which is the difference between epigenetic age and chronological age, has also been strongly associated with a wide-range of age-related diseases [7] as well as life expectancy across racial groups [8]. Numerous measures of EAA have been developed, each measuring unique aspects of the aging process, and include, among others: intrinsic EAA (IEAA) [9], which reflects aging independent of blood cell-type composition; HannumAge [10] acceleration (HannumAA), which is more reflective of extrinsic aging; and ‘second generation’ predictors like PhenoAge acceleration (PhenoAA) [11] and GrimAge acceleration (GrimAA) [12], which are built to better predict age-related diseases and mortality. In a recent multi-ethnic cross-sectional study, Matías-García et al. identified robust associations between kidney traits and various EAA measures in whole blood [13]. While these findings provide compelling support for a link between EAA and kidney function, the temporality of the relationship remains unclear and confounding inherent to the observational study design cannot be ruled out.

**Fig. 1:**
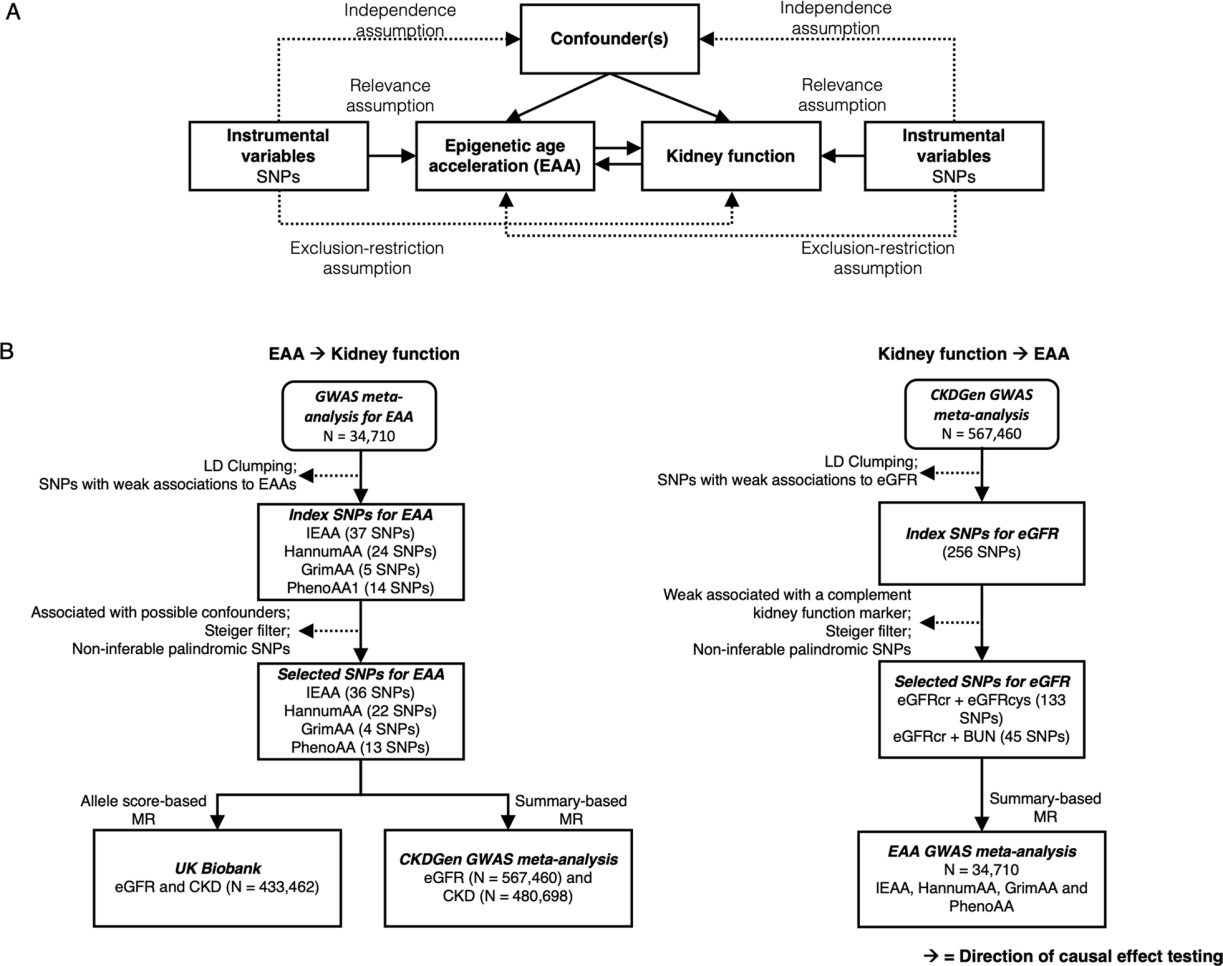
Study design diagram. **A** Mendelian randomization (MR) assumptions and hypothesized bidirectional relations between EAA and kidney function. **B** Flow diagram of the two-sample bidirectional MR study. *CKD* Chronic kidney disease; *eGFR* Estimated glomerular filtration rate; *GWAS* Genome-wide association study; *LD* Linkage disequilibrium; *SNP* Single nucleotide polymorphism

Mendelian randomization (MR) helps to support causal inference by leveraging genetic variants as instrumental variables for an exposure, with random genotype allocation mimicking intervention allocation in randomized controlled trials (RCTs) [14]. For complex traits, like EAA, MR studies utilize genetic instruments comprised of variants attaining genome-wide significance in large-scale genome-wide association studies (GWAS). With a GWAS meta-analysis of EAA just recently published [15], we now have the opportunity to explore the potentially causal association of EAA with kidney function and CKD risk. In this study, we leverage summary statistics from large-scale GWAS meta-analyses of EAA (*n* = 34,710) and kidney function (*n* = 567,460), as well as individual-level genotype and kidney function data from 433,462 White British UK Biobank participants, to carry-out the first MR study of EAA and kidney function phenotypes (Fig. 1). Our study is also one of only two MR studies relating biological aging more generally to CKD [16].

## Results

### Summary-level MR of EAA on kidney function

**Fig. 2:**
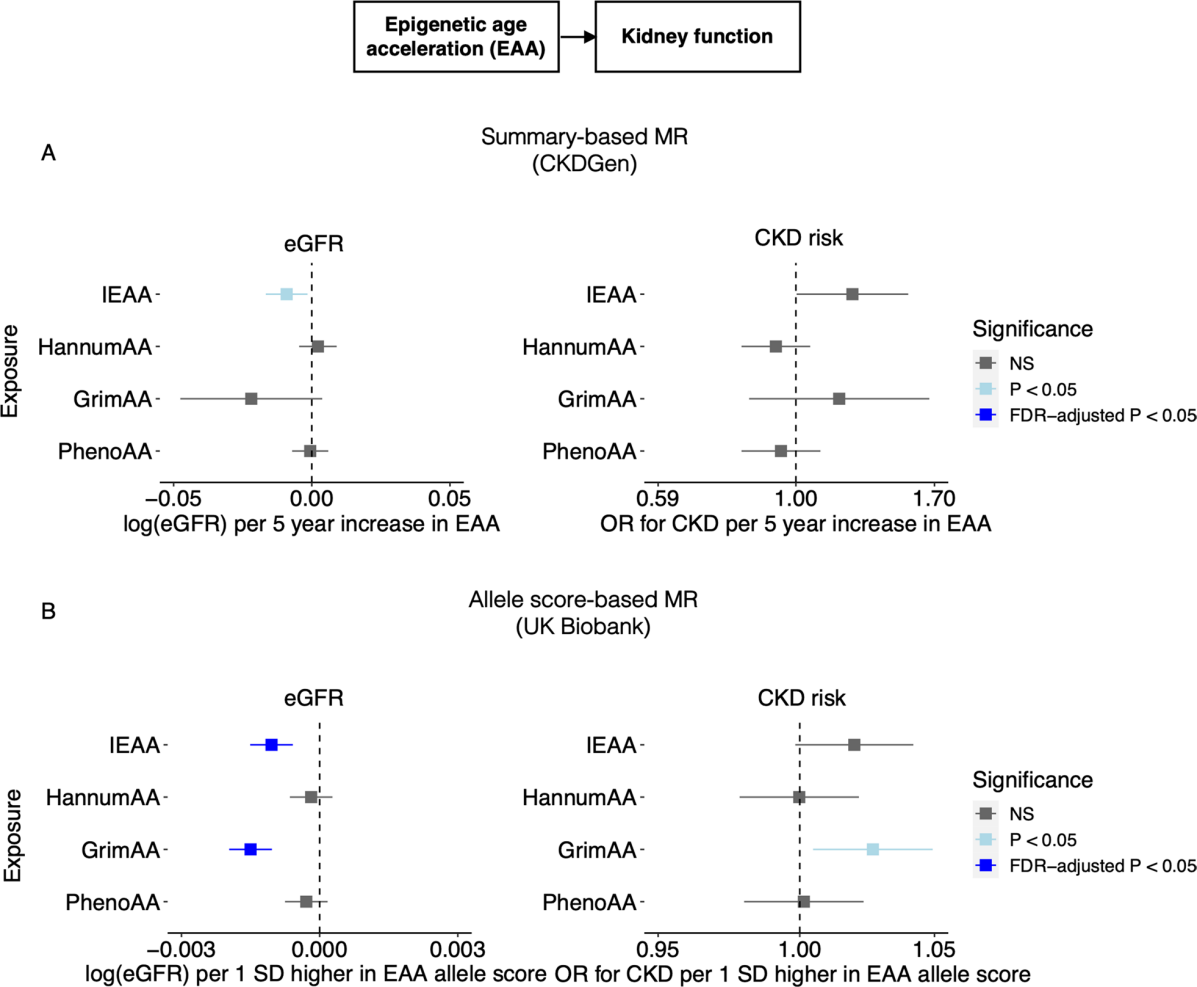
Causal estimates from genetically predicted EAA to eGFR and CKD. **A** Summary-based MR with CKDGen data. **B** Individual-level allele score-based MR with UK Biobank data. *CKD* Chronic kidney disease; *eGFR* Estimated glomerular filtration rate; *FDR* False discovery rate; *NS* Not significant; *OR* Odds ratio; *SD* Standard deviation

In the summary-level MR analysis, significant and marginally significant associations were identified between genetically predicted EAA and both eGFR and CKD (Fig. 2A, Table 1). A higher genetic predisposition to IEAA was significantly associated with a lower eGFR (*P* = 0.02), and marginally associated with a higher CKD risk (*P* = 0.051). The estimated effects were equivalent to a 0.01 decrease in log-transformed eGFR and a 24% increase in CKD risk per 5 year increase in IEAA. These causal estimates were based on the MR-Egger method, since significant directional pleiotropy was determined by the Rücker’s model-selection framework. However, these associations did not attain significance after FDR correction. After further excluding SNPs with relatively weak IEAA associations but potential pleiotropic effects, causal estimates remained consistent (Table 1, Additional file 1: Fig. S1). Sensitivity analyses using pleiotropy-robust methods, including the weighted median and MR-PRESSO approaches, were consistent in effect direction with the primary method but not statistically significant (Additional file 1: Tables S7 and S8). Genetically predicted GrimAA showed marginally significant causal associations with decreased eGFR (*P* = 0.09). Based on the IVW method, each 5 year increase in GrimAA was associated with an 0.02 decrease in log-transformed eGFR. This is supported by a series of sensitivity analyses using a conservative genetic instrument (Table 1, Additional file 1: Fig. S1) as well as pleiotropy-robust methods, where similar effect sizes with at least marginal significance in both weighted median and MR-PRESSO analyses were observed (Additional file 1: Table S7 and S8). No associations were observed between GrimAA and CKD in the main analysis. Similarly, causal estimates from PhenoAA and HannumAA were also nonsignificant. No indication of weak instrument strength was detected based on F-statistics for IVW analyses and *I*^2^ statistics for MR-Egger analyses (Additional file 1: Tables S7 and S8). Additional leave-one-out and single-SNP analyses did not identify any SNPs with disproportionate effects on the causal estimates (Additional file 1: Figs. S2–S5).

**Table 1:** Findings of summary-level MR investigating effects of EAA on eGFR and CKD

Genetically predicted exposure	Main analysis					Sensitivity analysis^a^				
	Genetic instruments	Egger intercept *P*	Primary method	Effect (95% CI)^b^	*P*	Genetic instruments	Egger intercept *P*	Primary method	Effect (95% CI)^b^	*P*
*eGFR*										
IEAA	36 SNPs	**0.02**	MR Egger	**− 0.01 (− 0.02, − 0.002)**	**0.02**^**c**^	22 SNPs	**0.07**	MR Egger	**− 0.01 (− 0.02, − 0.001)**	**0.03**^**c**^
HannumAA	22 SNPs	0.72	IVW	0.002 (− 0.005, 0.01)	0.52	19 SNPs	**0.06**	MR Egger	− 0.01 (− 0.02, 0.003)	0.20
GrimAA	4 SNPs	0.51	IVW	− 0.02 (− 0.05, 0.004)	0.09	3 SNPs	0.18	IVW	**− 0.03 (− 0.06, − 0.005)**	**0.02**^**c**^
PhenoAA	13 SNPs	0.92	IVW	− 0.001 (− 0.01, 0.01)	0.86	9 SNPs	0.39	IVW	− 0.003 (− 0.01, 0.005)	0.45
*CKD*										
IEAA	36 SNPs	**0.05**	MR Egger	1.24 (1.00, 1.54)	0.05	22 SNPs	**0.04**	MR Egger	**1.29 (1.06, 1.58)**	**0.02**^**c**^
HannumAA	22 SNPs	0.10	IVW	0.93 (0.81, 1.06)	0.25	19 SNPs	0.15	IVW	0.95 (0.86, 1.06)	0.35
GrimAA	4 SNPs	0.33	IVW	1.18 (0.84, 1.67)	0.35	3 SNPs	0.55	IVW	**1.36 (1.02, 1.80)**	**0.03**^**c**^
PhenoAA	13 SNPs	0.36	IVW	0.94 (0.81, 1.10)	0.46	9 SNPs	0.91	IVW	1.02 (0.87, 1.18)	0.84

*CI* Confident interval; *CKD* Chronic kidney disease; *eGFR* Estimated glomerular filtration rate; *SNP* Single nucleotide polymorphism; *IVW* Inverse variance weighted

^a^Sensitivity analysis using genetic instruments derived from a conservative set of variants after excluding SNPs with relatively weak EAA associations and strong associations with other phenotypes

^b^Effect sizes and CIs correspond to a 5 year increase in genetically predicted EAA. Effect sizes are presented as beta estimates for log-transformed eGFR and hazard ratios for CKD

^c^Nominally significant

### Allele score-based MR of EAA on kidney function

In the allele score-based MR analyses of UK Biobank data, each standard deviation increase in IEAA and GrimAA allele score (or genetically predicted IEAA or GrimAA) was significantly associated with a lower eGFR (*P* < 0.001, for both IEAA and GrimAA) and remained significant after False Discovery Rate (FDR) correction (Table 2). The results remained consistent after additionally adjusting clinical covariates, including body mass index (BMI), hypertension, diabetes, hypercholesterolemia, triglycerides, high-density lipoprotein cholesterol, low-density lipoprotein cholesterol, and smoking (Table 2, Fig. 2B). For the CKD endpoint, genetically predicted IEAA and GrimAA were marginally and significantly associated, respectively, with a higher risk of CKD in the clinical covariate-adjusted models (IEAA, *P* = 0.07; GrimAA, *P* = 0.02). The causal estimates for genetically predicted HannumAA and PhenoAA on kidney function outcomes remained nonsignificant. It should be noted that the estimates of association represent the difference in eGFR or hazard ratio for CKD per standard deviation increase in transformed allele scores, which explain relatively small proportions of the variation in their corresponding EAA measures. As expected, the presented effect sizes are small and do not reflect the magnitude of association between EAA itself and kidney function.

**Table 2:** Findings of individual-level allele score-based MR investigating causal effects of EAA on kidney function and CKD

Genetically predicted exposure	Model 1^a^		Model 2^b^	
	Effect (95% CI)^c^	*P*	Effect (95% CI)^c^	*P*
*eGFR*^d^				
IEAA	− **0.001 (**− **0.001, **− **0.001)**	**< 0.001**^**f**^	− **0.001 (**− **0.002, **− **0.001)**	**< 0.001**^**f**^
GrimAA	− **0.001 (**− **0.002, **− **0.001)**	**< 0.001**^**f**^	− **0.001 (**− **0.002, **− **0.001)**	**< 0.001**^**f**^
HannumAA	− 0.0001 (− 0.001, 0.0004)	0.76	− 0.0002 (− 0.001, 0.0003)	0.44
PhenoAA	− 0.0002 (− 0.001, 0.0002)	0.3	− 0.0003 (− 0.001, 0.0002)	0.22
*CKD*^d^				
IEAA	1.02 (1.00, 1.04)	0.10	1.02 (1.00, 1.04)	0.07
GrimAA	1.02 (1.00, 1.04)	0.13	**1.03 (1.00, 1.05)**	**0.02**^**e**^
HannumAA	1.00 (0.98, 1.02)	0.91	1.00 (0.98, 1.02)	0.99
PhenoAA	1.00 (0.98, 1.02)	0.78	1.00 (0.98, 1.02)	0.89

*CI* Confident interval; *CKD* Chronic kidney disease; *eGFR* Estimated glomerular filtration rate

^a^Adjusted for age, sex, and 10 ancestry principal components

^b^Adjusted for covariables in model 1 + hypertension, diabetes mellitus, lipid profiles, hypercholesterolemia, body mass index (BMI), and smoking

^c^Effect sizes and CIs correspond to a 1 Z-score increase in EAA allele score (or genetically predicted EAA). Effect sizes are presented as beta estimates for log-transformed eGFR and hazard ratios for CKD

^d^eGFR is based on the combined cystatin C/creatinine-based estimation using CKD-EPI equation. CKD stage 3–5 is defined by eGFR < 60 ml/min per 1.73 m^2^

^e^Nominally significant

^f^Significant after FDR correction

### Summary-level MR of kidney function on EAA

**Fig. 3:**
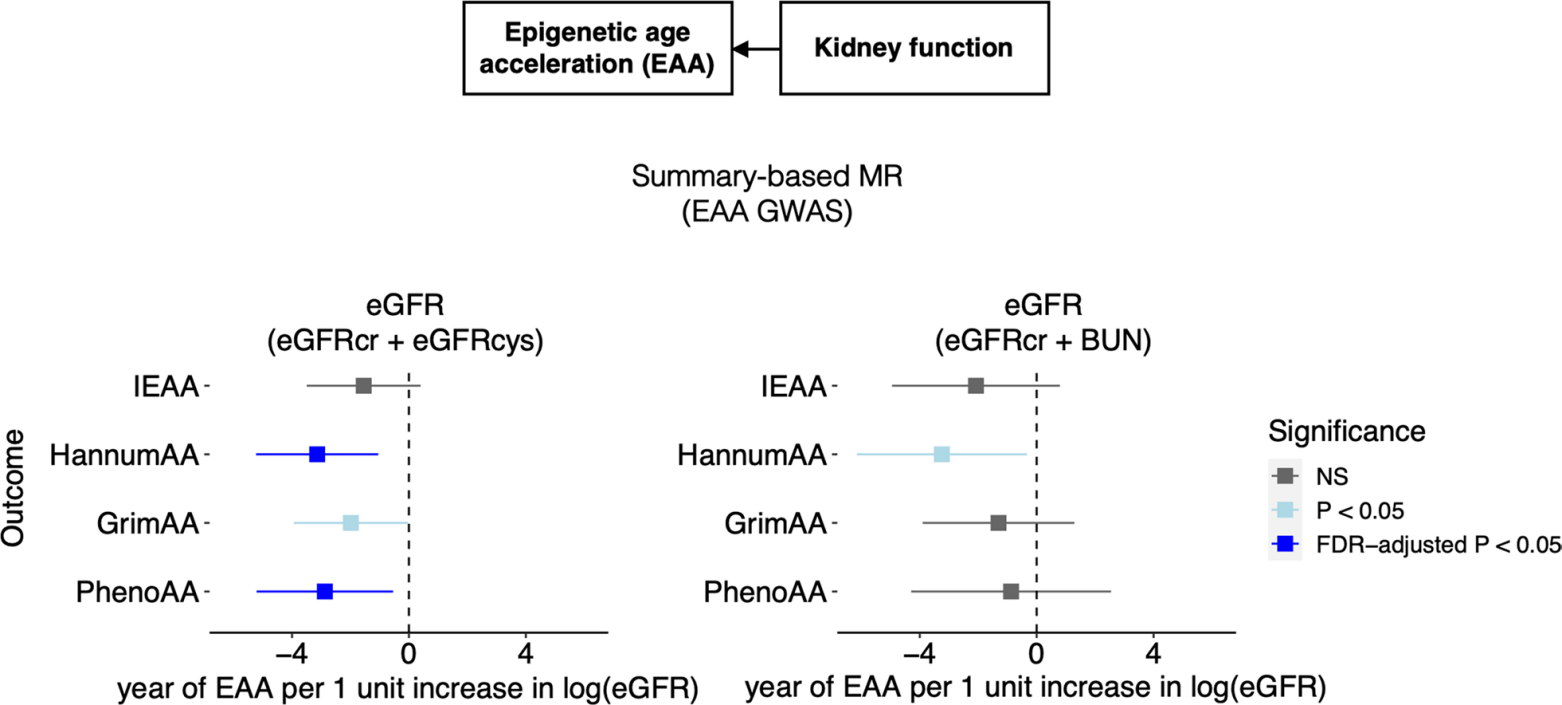
Causal estimates from genetically predicted eGFR to EAA. Summary-based MR with data from the GWAS meta-analysis of EAA. *eGFR* Estimated glomerular filtration rate; *FDR* False discovery rate; *NS* Not significant

Results from the reverse-direction summary MR analyses demonstrated a significant causal association between genetic predisposition to decreased kidney function and increased EAA (Table 3, Fig. 3). Using the eGFR_cr_ + eGFR_cys_ genetic instrument, each 1 unit higher log-transformed eGFR associated with a 3.14 year decrease in HanuumAA (*P* = 0.003), 1.99 year decrease in GrimAA (*P* = 0.05), and 2.88 year decrease in PhenoAA (*P* = 0.02). The association with HannumAA and PhenoAA remained significant after FDR correction. Notably, using the eGFR_cr_ + BUN genetic instrument, which was comprised of only 45 SNPS, elevated.

**Table 3:** Findings of summary-level MR investigating effects of eGFR on EAA

Outcome	Main analysis					Sensitivity analysis^a^				
	Genetic instruments	Egger intercept *P*	Primary method	Effect (95% CI)^b^	*P*	Genetic instruments	Egger intercept *P*	Primary method	Effect (95% CI)^b^	*P*
*eGFR (eGFR*_*cr*_ + *eGFR*_*cys*_*)*^c^										
IEAA	133 SNPs	0.91	IVW	− 1.55 (− 3.50, 0.40)	0.12	119 SNPs	0.78	IVW	− 1.57 (− 3.66, 0.52)	0.14
HannumAA	133 SNPs	0.23	IVW	− **3.14 (**− **5.23, **− **1.05)**	**0.003**^**e**^	119 SNPs	0.24	IVW	− **2.78 (**− **4.99, **− **0.57)**	**0.01**^**e**^
GrimAA	133 SNPs	0.17	IVW	− **1.99 (**− **3.94, **− **0.04)**	**0.05**^**d**^	119 SNPs	0.40	IVW	− 1.79 (− 3.87, 0.28)	0.09
PhenoAA	133 SNPs	0.40	IVW	− **2.88 (**− **5.21, **− **0.54)**	**0.02**^**e**^	119 SNPs	0.73	IVW	− **2.83 (**− **5.28, **− **0.37)**	**0.02**^**e**^
*eGFR (eGFR*_*cr*_ + *BUN)*^c^										
IEAA	45 SNPs	0.98	IVW	− 2.08 (− 4.95, 0.79)	0.16	39 SNPs	0.83	IVW	− 1.99 (− 5.06, 1.07)	0.20
HannumAA	45 SNPs	0.35	IVW	− **3.24 (**− **6.15, **− **0.34)**	**0.03**^**d**^	39 SNPs	0.37	IVW	− **3.27 (**− **6.43, **− **0.11)**	**0.04**^**d**^
GrimAA	45 SNPs	0.53	IVW	− 1.31 (− 3.90, 1.29)	0.32	39 SNPs	0.52	IVW	− 1.53 (− 4.28, 1.22)	0.28
PhenoAA	45 SNPs	0.90	IVW	− 0.88 (− 4.29, 2.54)	0.62	39 SNPs	0.81	IVW	− 0.59 (− 4.24, 3.06)	0.75

*BUN* Blood urea nitrogen; *CI* Confident interval; *eGFR* Estimated glomerular filtration rate; *SNP* Single nucleotide polymorphism; *IVW* Inverse variance weighted

^a^Sensitivity analysis using genetic instruments derived from variants after excluding SNPs with relatively weak EAA associations and significant pleiotropic effects with known confounders

^b^Effect sizes were from genetically predicted 1 unit increase in log-transformed eGFR to year in EAA

^c^Genetic instrument for kidney function is developed from a GWAS for creatinine-based eGFR, with an additional filter to only keep SNPs that are associated with a second marker of kidney function (cystatin C-based eGFR or BUN) after Bonferroni correction

^d^Nominally significant

^e^Significant after FDR correction

HannumAA remained significantly associated with a lower eGFR [beta (95% confidence interval) = − 3.24 (− 6.15, − 0.34), *P* = 0.03]. Non-significant, but directionally consistent estimates between the eGFR_cr_ + BUN genetic instrument and both GrimAA and PhenoAA and were observed. There was no causal association observed between kidney function and IEAA. Sensitivity analyses using conservative genetic instruments yielded generally consistent results with the main analysis. Sensitivity analyses using MR-Egger, weighted median and MR-PRESSO showed similar effect estimation with a few exceptions to MR-Egger, which has low statistical power in the absence of horizontal pleiotropy (Egger intercept *P* > 0.1) (Additional file 1: Tables S9 and S10). No indication of weak instrument strength was detected based on F-statistics for IVW analyses and I^2^ statistics for MR-Egger analyses (Additional file 1: Tables S9 and S10). Further, an exploratory multivariable MR was conducted to assess the potential mediating role of lymphocyte count on the identified causal association between kidney function and HannumAA. Leveraging a summary-level multivariable MR, we identified that the lymphocyte count mediates 38% of the total effect of eGFR on HannumAA [indirect effect − 1.19, 95% CI (1.65, − 4.03), Additional file 1: Table S11].

### Power calculations for the summary-level MR of EAA on kidney function

In light of the lack of significant associations from our summary-level MR of EAA on kidney function, we assessed the statistical power of this two-sample analysis. Our MR power analysis indicated that all EAA measures, except for IEAA, had insufficient power to detect less than a 15–25% increased odds of CKD per 1 SD increase in EAA (Additional file 1: Table S12).

## Discussion

In the first large-scale MR study to investigate associations between epigenetic aging and kidney phenotypes, we identified causal bidirectional relationships between EAA and kidney function. Among four measures of EAA examined, genetically increased IEAA was significantly associated with decreased eGFR and marginally associated with increased CKD risk, findings which were consistent across CKDGen and UK Biobank and sensitivity analyses using conservative genetic instruments. In addition, increased GrimAA significantly associated with decreased eGFR and increased risk of CKD in UK Biobank, with similar but non-significant trends observed in CKDGen. Interestingly, increased GrimAA may also result from decreased kidney function, as demonstrated by our reverse-direction MR analysis. These findings indicate that GrimAA could be both a cause and consequence of kidney function decline. Reverse-direction MR study further supported causal effects of decreased kidney function on HanumAA and PhenoAA. Our results may have important clinical and public health implications. There is growing evidence suggesting that DNA methylation may be an actionable target for disease prevention and treatment [3, 17], with a recent small non-randomized intervention trial demonstrating the reversal of EAA after twelve months administration of recombinant human growth hormone, dehydroepiandrosterone, and metformin [18]. Taken together, these findings point to potential therapeutic strategies that could be prioritized for the prevention of kidney function decline in high risk populations with increased EAA. Furthermore, our findings of kidney function decline preceding increased EAA implicate molecular mechanisms that may link CKD to its sequelae.

We are the first study to report a significant and potentially causal effect of IEAA on kidney function. Derived from Horvath’s clock for age acceleration after regressing out blood cell estimates, IEAA is known as one of the most robust biological aging measures across cell types and organs [8, 9]. In previous studies, IEAA has exhibited a lack of association with lifestyle risk factors [19–21] but has been strongly and reproducibly associated with all-cause mortality [8, 9, 21, 22]. Notably, IEAA was highly correlated with age-related clonal haemopoiesis [23], a known manifestation of the cellular aging process [21, 24]. Very few studies have examined associations of IEAA with kidney function [13, 25]. One previous observational study of 1389 Black participants found no association between IEAA and eGFR but observed a significant association of IEAA with urinary albumin to creatinine ratio [25]. In addition, Matías-García et al. identified nominally significant cross-sectional associations between IEAA and eGFR in their recent multi-ancestry meta-analyses [13]. Our study adds more robust evidence of association to this literature, with significant associations observed across the large CKDGen and UK Biobank cohorts. We present further evidence of a temporal, causal relationship between IEAA and kidney function. Given the close tie between IEAA and cellular aging [21, 23, 24], our finding suggests a causal pathway that may link accelerated biological age to reduced kidney function and, potentially, development of CKD. Still, mechanistic understanding of how the intracellular methylome-level changes that determine IEAA affect downstream gene expression and subsequently lead to adverse effects on kidney health demands additional study.

We also reported potentially causal bidirectional associations between GrimAA and kidney function. While we note caution in the interpretation of these results, given the small number of variants included in our GrimAA genetic instrument, our findings are consistent with the only previous study to examine this association [13]. In the report by Matías-García, marginally significant cross-sectional associations of GrimAA with decreased eGFR and increased CKD risk were identified [13]. Although limited data exist on the bidirectional relationship between GrimAA and kidney endpoints, numerous studies have identified associations between GrimAA and CKD risk factors, such as smoking, adiposity, lipids, blood pressure, and fasting plasma glucose [12, 15, 26]. Furthermore, adiposity-related phenotypes have been temporally and potentially causally associated with GrimAA [15], which in combination with our findings, suggest that GrimAA could mediate the associations between lifestyle risk factors and kidney function. Hence, our findings provide novel mechanistic hypotheses for future studies [20].

Like GrimAA, decreased kidney function was observed to have increasing effects on HannumAA and PhenoAA in our reverse MR analyses. In the only previous study to examine this relationship, Matías-García et al. observed strong cross-sectional associations of both of these measures with all kidney function phenotypes examined, including eGFR, CKD, urinary albumin to creatinine ratio, and microalbuminuria [13]. Given the significant associations observed in our reverse MR study, combined with the lack of an effect of both HannumAA and PhenoAA on kidney function in our primary MR analysis, our data suggest that kidney function decline may temporally precede EAA, as measured by HannumAA and PhenoAA. HannumAA and PhenoAA are both considered extrinsic measures of epigenetic aging due to their association to with age-related cell composition shifts [7]. Intriguingly, a previously conducted MR analysis demonstrated causal effects of low lymphocyte counts on HannumAA [15]. Because low lymphocytes have been previously associated with CKD and its progression [27, 28], these aggregate data suggest a potential link between damaged glomerular function and exacerbated aging of blood and immune cells that could mechanistically result from lymphocyte depletion. The results from our exploratory multivariable MR analysis indicate that lymphocyte count mediates the effect of eGFR on HannumAA, a finding that is consistent with existing literature [15, 27, 28] and provides novel insights into the mechanisms linking renal function and biological aging.

Our study has several technical and conceptual strengths. This is the first study to investigate bidirectional relationships between the EAA and kidney function, providing temporal evidence to better articulate the associations observed in previous cross-sectional studies. Moreover, we leveraged large-scale datasets, including summary statistics from two GWAS meta-analyses comprised of up 567,460 participants and individual-level data from 433,462 UK Biobank participants. In addition, our primary analyses investigating effects of EAA on kidney function phenotypes were examined for reproducibility across the two independent cohorts using distinct approaches that included summary-based analyses and more powerful, allele score-based methods. Furthermore, we employed a variety of techniques and sensitivity analyses to assess the robustness of our findings under various MR assumptions. Some limitations of this study also warrant mentioning. Although we used the largest GWAS meta-analysis of EAA to develop our genetic instruments [29], the GrimAA genetic instrument included only 4 SNPs that explained less than 1% of the variability in GrimAA. Therefore, careful interpretation of our findings is recommended. While other genetic instruments contained more SNPs and explained up to 4% of the variability in their corresponding EAA measure, which is consistent with variances explained for other complex phenotypes that have been examined in MR study [30, 31], nonsignificant results should be interpreted cautiously and may not necessarily support an absence of causal relationships [29]. Indeed, based on our power calculations, the lack of significant associations of EAA on CKD risk may be due to limited power to detect relatively small effects. Besides, the CKDGen data used for the summary-based MR analysis have some overlap with the GWAS meta-analysis of EAA, which may cause bias toward associations reported by observational studies [32]. However, results of the summary-based MR were generally consistent with that of the UK Biobank, which leveraged an entirely independent dataset. Furthermore, because the previous GWAS were conducted exclusively in participants of European ancestry, the generalizability of these findings may be limited. Future studies in more diverse populations are critically necessary.

## Conclusion

In summary, our two-sample MR study provides evidence of bidirectional causal relationships between methylation-based age acceleration and kidney function phenotypes. Our results suggest that IEAA and GrimAA may causally relate to decreased kidney function, which in turn could further increase GrimAA, HannumAA, and PhenoAA, creating a positive feedback loop where biological aging begets kidney function decline which further exacerbates biological aging. Furthermore, given that EAA has been linked to numerous cardiometabolic diseases and clinical CVD [20, 26, 33], EAA could play an important role in the higher frequency of these conditions observed in the CKD setting. Future research to further articulate molecular mechanisms of EAA and their role in the development of CKD and its sequelae are needed.

## Methods

### Study design overview

In this study, two-sample MR analyses were performed to investigate causal effects of EAA on kidney function, and vice versa (Fig. 1A). When modeling EAA as exposure, genetic instruments for four EAA measures, namely IEAA, HannumAA, GrimAA and PhenoAA, were implemented based on the most-recent large-scale GWAS meta-analyses of EAAs among individuals of European-ancestry [15]. Summary-based MR was conducted using summary statistics from a GWAS meta-analysis of estimated glomerular filtration rate (eGFR) based on participants of European-ancestry in the CKDGen Consortium [34]. Additionally, allele score-based MR was performed leveraging individual-level genotype and kidney function data available from the UK Biobank [35] in a one-sample setting. When modeling kidney function as exposure, genetic instruments for log-transformed eGFR values were derived from the CKDGen Consortium’s GWAS meta-analysis of kidney function and used to perform summary-based MR utilizing the summary statistics from the GWAS meta-analysis of EAA (Fig. 1B).

### MR assumptions

MR analysis requires three core assumptions that define valid instrumental variables, as shown in Fig. 1A [36]. (i) The ‘relevance assumption’ requires that the genetic instrument must be associated with the exposure phenotype. (ii) The ‘independence assumption’ requires that the genetic instrument should be independent of confounders. (iii) The ‘exclusion-restriction assumption’ requires that the genetic instrument must be associated with the outcome through the exposure phenotype only.

To satisfy the ‘relevance assumption’, genetic instruments for EAA or kidney function were restricted to genetic variants attaining genome-wide significance in GWAS meta-analysis. To meet the ‘independence assumption’, SNPs associated with known confounders were removed from genetic instruments. SNPs with any potential pleiotropic effects were further excluded in sensitivity analyses. Further, multiple pleiotropy-robust MR approaches that can relax the ‘independence’ and ‘exclusion-restriction’ assumptions were applied as additional sensitivity analyses (Additional file 1: Supplementary Methods).

### Selection of genetic instruments

Genetic instruments for four distinct EAA measures, i.e., IEAA, HannumAA, GrimAA and PhenoAA, were derived from a recent GWAS meta-analysis for epigenetic aging [15]. As the largest GWAS of EAA to date, the meta-analysis included 34,710 ancestrally European participants from 28 cohorts. Genetic instruments for each EAA variable were comprised of independent SNPs (500-kb window, *r*^2^ < 0.1) that achieved genome-wide significant associations (*P* = 5 × 10^–8^) with EAA in GWAS meta-analysis, with exclusion of SNPs robustly associated with hypertension, blood pressure, diabetes mellitus, cholesterol-lowering medications, body mass index (BMI), obesity, and smoking (described in detail the Additional file 1: Supplementary Methods). Among SNPs selected for the EAA genetic instruments, no ambiguous and non-inferable palindromic SNPs were found. The Steiger filtering [37], a procedure that removes SNPs failing to explain significantly more variance in the exposure than in the outcome, was performed to confirm the directionality of instrument SNPs. Summary statistics for the IEAA (36 SNPs), HannumAA (22 SNPs), PhenoAA (13 SNPs), and GrimAA (4 SNPs) genetic instruments are presented in Additional file 1: Tables S1–S4. Effect sizes from GWAS summary statistics were aligned toward “increasing” EAA. In sensitivity analyses assessing whether causal estimates were affected by the inclusion of potentially pleiotropic variants [36], more conservative genetic instruments for each EAA variable were implemented. In these analyses, Phenoscanner V2.0 was used to further remove SNPs with any reported phenotype association [38] (see Additional file 1: Supplementary Methods and Tables S1–S4 for details).

To meet the ‘relevance criteria’ and avoid the development of a genetic instrument reflecting creatinine metabolism rather than kidney function alone [16, 30, 31], two genetic instruments for kidney function were developed. Both were derived from 256 index SNPs achieving genome-wide significance (*P* = 5 × 10^–8^) with log-transformed serum creatinine-based eGFR in the large-scale CKDGen GWAS meta-analysis conducted in a predominantly ancestrally European population. From there, SNPs further demonstrating weak or no associations with cystatin C-based eGFR or blood urea nitrogen (BUN) were removed, separately, leaving 140 SNPs as the basis for a creatinine-based eGFR plus cystatin-C-based eGFR (eGFR_cr_ + eGFR_cys_) genetic instrument and 47 SNPs for a creatine-based eGFR plus BUN (eGFR_cr_ + BUN) genetic instrument, respectively. After performing additional filtering steps including a confirmative clumping, removal of palindromic SNPs with intermediate allele frequency, and Steiger filtering, 133 and 45 SNPs remained in the respective eGFR_cr_ + eGFR_cys_ and eGFR_cr_ + BUN genetic instruments for kidney function (Additional file 1: Tables S5 and S6). In sensitivity analyses, genetic instruments further removing SNPs significantly associated with potential confounding factors, namely BMI, obesity and smoking, were implemented.

To ensure the validity of bidirectional MR, genetic instruments for exposures in this bidirectional analysis, namely EAA and kidney function, were examined to make sure they were independent from each other. First, instrumental SNPs for exposures in the bidirectional analysis were examined, as suggested by Smith et al*.* [39]. We identified no overlapping SNPs or SNPs in high LD. Second, when removing confounder-associated SNPs (including SNPs in high LD with confounders), we also searched for all exposure-related SNPs in this bidirectionally analysis. This was performed using the PhenoScanner v2.0, following the same approach as we described for the removal of confounder-associated SNPs, and SNPs in high LD (*r*^2^ > 0.8) them. As a result, rs1598856, a SNP in genetic instrument for HannumAA, was found in strong association with eGFR at genome-wide significance in GWAS studies. This SNP was excluded by the PhenoScanner filter in the sensitivity analysis as shown in Supplemental Table S2, and the removal of this SNP did not significantly affect the results. No additional SNPs were identified.

### Outcome data for two-sample summary-based MR

To investigate the effects of our EAA genetic instruments on kidney function outcomes, we used summary statistics from the CKDGen Consortium (*N* = 567,460 for eGFR; *N* = 480,698 for CKD, including 41,395 CKD cases). Summary statistics for kidney function traits, including log-transformed eGFR and incident CKD (eGFR < 60 ml/min per 1.73 m^2^), were downloaded from the CKDGen Consortium public domain website (https://ckdgen.imbi.uni-freiburg.de) and used as outcome data. To investigate the effects of our kidney function genetic instruments on the EAA outcomes of interest, we used summary statistics from the most-recent large-scale GWAS meta-analyses of EAA (*N* = 34,710 for all EAA measures), which were downloaded from https://datashare.is.ed.ac.uk/handle/10283/3645 [15].

### Outcome data for allele score-based MR

The UK Biobank is a prospective cohort of more than 500,000 individuals aged 50 to 65 years who have been examined using a standard protocol at multiple sites throughout the United Kingdom [35]. In this study, we included 433,462 White British UK Biobank participants that passed standard quality filters [40] (Additional file 1: Supplementary Methods) and had measured serum cystatin C and creatinine values at baseline. eGFR value was calculated using the CKD-EPI creatinine–cystatin equation [41], and CKD was defined as an eGFR < 60 ml/min per 1.73 m^2^.

### Summary-based MR to investigate causal effects of EAA on kidney function phenotypes and kidney function on EAA phenotypes

For summary-level MR, the Rücker model-selection framework [42, 43] was adopted to determine the primary MR method. Briefly, the multiplicative random-effects inverse variance weighted (IVW) method, which has the best power when all SNPs are valid instrumental variables [44], was used as the primary MR approach in the absence of significant horizontal pleiotropic effect. However, when horizontal pleiotropy was detected and the MR-Egger [45] represented as a better fit than IVW, the MR-Egger method was considered as the primary method and findings from this approach were reported. This model switching was determined based on a significant difference (*P* < 0.05) between Cochran’s Q statistic for the IVW method and Rücker’s Q’ for the MR-Egger method (with respect to a $\chi_{1}^{2}$ distribution), along with a significant nonzero MR-Egger intercept (*P* < 0.1) [42, 43]. For each analysis, we reported results from the Steiger test for directionality to indicate whether the directionality is valid. The strength of the genetic instrument for IVW and MR-Egger analyses was confirmed by measuring the F-statistic [46] and *I*^2^ statistic [47, 48], respectively. Further, additional methods that partially relax MR assumptions, including weighted median [49] and Mendelian Randomization Pleiotropy ReSidual Sum and Outlier (MR-PRESSO) [50], were performed as sensitivity analyses to further assess causality in the presence of unbalanced pleiotropy [36]. A detailed description of each method is provided in the Additional file 1: Supplementary Methods. To examine whether single SNPs were responsible for the causal associations observed, leave-one-out and single-SNP sensitivity analyses were also performed. Summary-level MR analyses were performed by TwoSampleMR [51] and MR-PRESSO [50] packages, and a 2-sided *P* < 0.05 was considered nominally significant. To account for multiple testing of four EAA measures, FDR correction was performed with an adjusted *P* < 0.05 considered significant. Original *P*-values were displayed in tables and figures throughout this study with footnotes indicating their significance after the FDR correction.

### Allele score-based MR of individual-level data to investigate causal effects of EAA on kidney function phenotypes

As a complement to summary-based MR analyses, individual-level allele score-based MR analyses were conducted by applying genetic instruments derived from the EAA GWAS meta-analysis to the UK Biobank using publicly available imputed genotype and phenotype data [40]. For each EAA measure, an allele score was calculated using PLINK software [52]. In brief, the allele score was calculated for each participant by multiplying the effect size of a SNP by a participant’s dosage of that same SNP for all SNPs comprising the genetic instrument, which included 23 SNPs for IEAA, 17 for HannumAA, 8 for PhenoAA, and 4 for GrimAA. Products were then summed across all SNPs in each EAA genetic instrument and standard normal transformed to create the allele scores. Multivariable linear and logistic regression models were used to test associations of each allele-score with eGFR and CKD, respectively, after adjustment for age, sex, and 10 ancestry principal components (Model 1). Additional clinical covariates, including hypertension, diabetes mellitus, lipid profiles, hypercholesterolemia, body mass index (BMI), and smoking, were adjusted in sensitivity analyses (Model 2).

### Power analysis

Statistical power analysis of MR was performed using the method proposed by Brion et al. [53]. Briefly, power estimates for detecting a causal effect of EAA on eGFR and CKD were calculated based on the sample size (and the proportion of cases for CKD, a binary outcome), variance explained (*R*^2^) for genetic instruments on exposure, true causal effect sizes, and a type-I error rate level (*α* = 0.05). Sample sizes were based on the CKDGen GWAS study. The average *R*^2^ for genetic instruments was estimated for each EAA based on the EAA GWAS meta-analysis [15]. True causal effect sizes were estimated based on double-standardized regression coefficients from the largest meta-analysis of EAA and kidney function published by Matías-García et al. [13]. The unit of exposure and outcome were standardized for the power calculation.

### Multivariable MR and mediation analysis

Multivariable MR and mediation analysis were applied to investigate whether the lymphocyte count mediates the associations of kidney function (approximated by eGFR) and HannumAA. The genetic instrument of lymphocyte counts was derived by extracting GWAS summary statistics of the Blood Cell Consortium [54] within the TwoSampleMR package [51]. Details for the derivation of the genetic instrument for eGFR (i.e., eGFRcr + eGFRcys) and outcome summary data for HannumAA were described in the corresponding Methods section. Genetic instruments were harmonized following the default procedure in the TwoSampleMR package. Multivariable MR was performed using the IVW method. The direct and indirect effects were calculated following the “difference method” [55], which leverages the univariate MR to estimate the total effect of eGFR on HannumAA and the multivariable MR to estimate the direct effect of the eGFR on the HannumAA conditional on the lymphocyte count.

## Supplementary Information

**Additional file 1: Fig. S1.** Sensitivity analyses of summary-based MR using a conservative genetic instrument from genetically predicted EAA to eGFR and CKD. **Fig. S2.** Single-SNP and leave-one-out analyses for the causal estimates from genetically predicted IEAA to eGFR and CKD. **Fig. S3.** Single-SNP and leave-one-out analyses for the causal estimates from genetically predicted GrimAA to eGFR and CKD. **Fig. S4.** Single-SNP and leave-one-out analyses for the causal estimates from the sensitivity analysis using a conservative genetic instrument from genetically predicted IEAA to eGFR and CKD. **Fig. S5.** Single-SNP and leave-one-out analyses for the causal estimates from the sensitivity analysis using a conservative genetic instrument from genetically predicted GrimAA to eGFR and CKD. **Table S1.** Summary of lead SNPs for the genetic instrument of IEAA. **Table S2.** Summary of lead SNPs for the genetic instrument of HannumAA. **Table S3.** Summary of lead SNPs for the genetic instrument of GrimAA. **Table S4.** Summary of lead SNPs for the genetic instrument of PhenoAA. **Table S5.** Summary of lead SNPs for the genetic instrument of kidney function based on serum creatinine eGFR refined by serum cystatin eGFR. **Table S6.** Summary of lead SNPs for the genetic instrument of kidney function based on serum creatinine eGFR refined by BUN. **Table S7.** Findings of summary-based MR from genetically predicted EAA to eGFR and CKD based on the primary method and pleiotropy or outlier-robust methods. **Table S8.** Findings of summary-based MR sensitivity analysis using conservative genetic instruments from genetically predicted EAA to eGFR and CKD based on the primary method and pleiotropy or outline-robust methods. **Table S9.** Findings of summary-based MR from genetically predicted eGFR to EAA based on the primary method and pleiotropy or outlier-robust methods. **Table S10.** Findings of summary-based MR sensitivity analysis using conservative genetic instruments from genetically predicted eGFR to EAA based on the primary method and pleiotropy or outline-robust methods. **Table S11.** Multivariable MR and mediation analysis of lymphocyte count on the association of kidney function with HannumAA. **Table S12.** Power analysis of the two-sample MR using the Brion et al. method.

## Data Availability

The present study was based on publicly available data. GWAS summary statistics are available at the CKDGen website (https://ckdgen.imbi.uni-freiburg.de/) and Edinburgh DataShare (https://datashare.is.ed.ac.uk/handle/10283/3645) and corresponding GWAS Catalog entries (accession numbers GCST90014287-GCST90014304). Individual-level genotype and phenotype data from UK Biobank Consortium are available at https://www.ukbiobank.ac.uk/data-showcase.
