# Supplementary material for: Effects of epigenetic age acceleration on kidney function: a Mendelian randomization study
Source: Clin Epigenetics. 2023 Apr 8;15:61. doi: 10.1186/s13148-023-01476-y (PMC10082992; doi:10.1186/s13148-023-01476-y)
Supplement: Supplementary file 1 — Additional file 1: Fig. S1. Sensitivity analyses of summary-based MR using a conservative genetic instrument from genetically predicted EAA to eGFR and CKD. Fig. S2. Single-SNP and leave-one-out analyses for the causal estimates from genetically predicted IEAA to eGFR and CKD. Fig. S3. Single-SNP and leave-one-out analyses for the causal estimates from genetically predicted GrimAA to eGFR and CKD. Fig. S4. Single-SNP and leave-one-out analyses for the causal estimates from the sensitivity analysis using a conservative genetic instrument from genetically predicted IEAA to eGFR and CKD. Fig. S5. Single-SNP and leave-one-out analyses for the causal estimates from the sensitivity analysis using a conservative genetic instrument from genetically predicted GrimAA to eGFR and CKD. Table S1. Summary of lead SNPs for the genetic instrument of IEAA. Table S2. Summary of lead SNPs for the genetic instrument of HannumAA. Table S3. Summary of lead SNPs for the genetic instrument of GrimAA. Table S4. Summary of lead SNPs for the genetic instrument of PhenoAA. Table S5. Summary of lead SNPs for the genetic instrument of kidney function based on serum creatinine eGFR refined by serum cystatin eGFR. Table S6. Summary of lead SNPs for the genetic instrument of kidney function based on serum creatinine eGFR refined by BUN. Table S7. Findings of summary-based MR from genetically predicted EAA to eGFR and CKD based on the primary method and pleiotropy or outlier-robust methods. Table S8. Findings of summary-based MR sensitivity analysis using conservative genetic instruments from genetically predicted EAA to eGFR and CKD based on the primary method and pleiotropy or outline-robust methods. Table S9. Findings of summary-based MR from genetically predicted eGFR to EAA based on the primary method and pleiotropy or outlier-robust methods. Table S10. Findings of summary-based MR sensitivity analysis using conservative genetic instruments from genetically predicted eGFR to EAA based on the pr [file 13148_2023_1476_MOESM1_ESM.docx]

**Additional Files Table of Contents**

**Figure S1.** Sensitivity analyses of summary-based MR using a conservative genetic instrument from genetically predicted EAA to eGFR and CKD.

**Figure S2.** Single-SNP and leave-one-out analyses for the causal estimates from genetically predicted IEAA to eGFR and CKD.

**Figure S3.** Single-SNP and leave-one-out analyses for the causal estimates from genetically predicted GrimAA to eGFR and CKD.

**Figure S4.** Single-SNP and leave-one-out analyses for the causal estimates from the sensitivity analysis using a conservative genetic instrument from genetically predicted IEAA to eGFR and CKD.

**Figure S5.** Single-SNP and leave-one-out analyses for the causal estimates from the sensitivity analysis using a conservative genetic instrument from genetically predicted GrimAA to eGFR and CKD.

**Supplementary Methods**

**Table S1.** Summary of lead SNPs for the genetic instrument of IEAA.

**Table S2.** Summary of lead SNPs for the genetic instrument of HannumAA.

**Table S3.** Summary of lead SNPs for the genetic instrument of GrimAA.

**Table S4.** Summary of lead SNPs for the genetic instrument of PhenoAA.

**Table S5.** Summary of lead SNPs for the genetic instrument of kidney function based on serum creatinine eGFR refined by serum cystatin eGFR.

**Table S6.** Summary of lead SNPs for the genetic instrument of kidney function based on serum creatinine eGFR refined by BUN.

**Table S7.** Findings of summary-based MR from genetically predicted EAA to eGFR and CKD based on the primary method and pleiotropy or outlier-robust methods.

**Table S8.** Findings of summary-based MR sensitivity analysis using conservative genetic instruments from genetically predicted EAA to eGFR and CKD based on the primary method and pleiotropy or outline-robust methods.

**Table S9.** Findings of summary-based MR from genetically predicted eGFR to EAA based on the primary method and pleiotropy or outlier-robust methods.

**Table S10.** Findings of summary-based MR sensitivity analysis using conservative genetic instruments from genetically predicted eGFR to EAA based on the primary method and pleiotropy or outline-robust methods.

**Table S11.** Multivariable MR and mediation analysis of lymphocyte count on the association of kidney function with HannumAA

**Table S12**. Power analysis of the two-sample MR using the Brion et al. method

**Supplementary Figures**


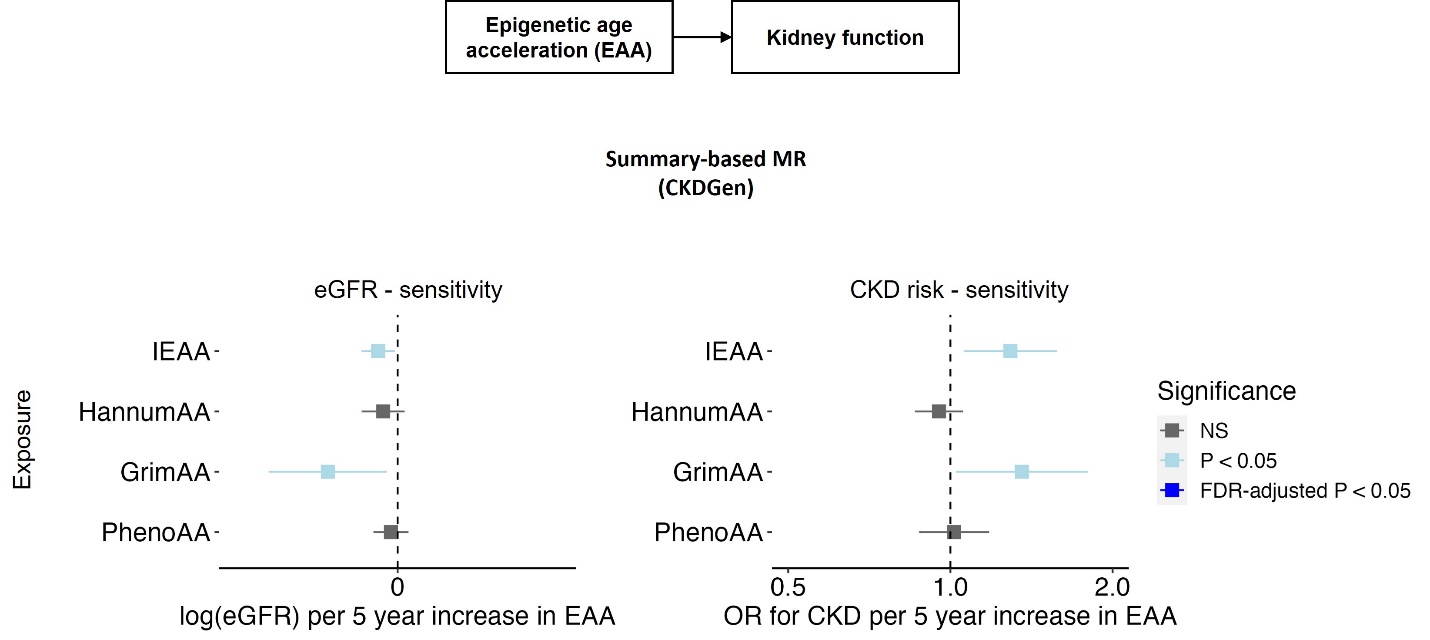


**Figure S1.** Sensitivity analyses of summary-based MR using a conservative genetic instrument from genetically predicted EAA to eGFR and CKD.

**Figure S2.** Single-SNP and leave-one-out analyses for the causal estimates from genetically predicted IEAA to eGFR and CKD.

**Figure S3.** Single-SNP and leave-one-out analyses for the causal estimates from genetically predicted GrimAA to eGFR and CKD.

**Figure S4.** Single-SNP and leave-one-out analyses for the causal estimates from the sensitivity analysis using a conservative genetic instrument from genetically predicted IEAA to eGFR and CKD.

**Figure S5.** Single-SNP and leave-one-out analyses for the causal estimates from the sensitivity analysis using a conservative genetic instrument from genetically predicted GrimAA to eGFR and CKD.

**Supplementary Methods**

**Pleiotropy-robust sensitivity analyses**

Previous literatures suggested to additionally perform a selection of robust methods with distinct assumptions as a sensitivity analysis to explore the validity of ‘independence’ and ‘exclusion-restriction’ assumptions in the conducted MR study [1]. Hence, in this study, we performed several commonly used robust MR methods, namely MR-Egger [2], weighted median [3] and MR-PRESSO [4].

The MR-Egger [2] regression is a Pleiotropy-robust method that estimates and adjusts for an overall pleiotropic effect on all SNVs. The estimated Egger intercept is also useful in the Rücker framework [5], which was used for the model section of primary MR approach in this study. MR-Egger provides consistent estimates of the causal effect under the Instrument Strength Independent of Direct Effect (InSIDE) assumption, which requires pleiotropic effects of genetic variants to be uncorrelated to genetic variant–exposure association. Weighted median [3] is also a pleiotropy-robust method that relaxes the requirement of valid instrumental SNPs. This method generates consistent estimates when majority, or more than half of the instrumental SNPs are valid. Mendelian Randomization Pleiotropy ReSidual Sum and Outlier (MR-PRESSO) is an outlier-robust MR method that detects and removes SNPs whose causal estimates statistically significantly different from other instrumental SNPs before performing final causal estimation.

**Excluding SNPs associated with potential confounders for EAA genetic instruments**

Genetic instruments for each EAA variable were comprised of independent SNPs with EAA in GWAS meta-analysis, with exclusion of SNPs associated with potential confounders. These confounders include hypertension, blood pressure, diabetes mellitus, cholesterol-lowering medications, body mass index (BMI), obesity, and smoking. To implement such filter, summary statistics of GWAS analysis results of corresponding traits based on UK Biobank were retrieved from an external resource available at https://yanglab.westlake.edu.cn/data/ukb_fastgwa/imp/. EAA index SNPs that had a genome-wide significant association (P=5×10^–8^) with potential confounders were removed. This external GWAS was conducted using fastGWA [6], and adjusted for age, sex, and the first 10 genetic principal components within the individual-level UK Biobank data.

**Excluding SNPs associated with other phenotypes for conservative EAA genetic instruments**

For sensitivity analysis from EAA to kidney function, conservative genetic instrument for each EAA SNPs achieved genome-wide significance (P=5×10^–8^) with any non-EAA traits identified in Phenoscanner V2.0 [7] were discarded, except for the top 3 SNPs with the strongest association to the corresponding EAA in the meta-GWAS (this exception is applied to 2 SNPs in PhenoAA, rs1142345, rs7228835; and 3 SNPs in GrimAA, rs9386796, rs887466, rs4065321). This led to excluding of 14 additional SNPs for IEAA, 3 for HanumAA, 1 for GrimAA and 4 for PhenoAA as shown in **Supplementary Table S1-4**.

**UK Biobank genotype and sample quality control**

Standard quality control steps were applied to UK Biobank imputed genotype data set. Variants were filtered out for low imputation quality R^2^ (<0.3), missing genotype (>0.01), low minor allele frequency (<0.01) and Hardy-Weinberg equilibrium (<1×10^-9^) using PLINK software [8]. Then, white British UK Biobank participants that passed standard quality filters, including missing rate, excessive heterozygosity, were included in the analysis. In total, 433,462 individuals with measured serum cystatin C and creatinine values at baseline were included for this study.

**Reference**

1. Burgess S, Smith GD, Davies NM, Dudbridge F, Gill D, Glymour MM, et al. Guidelines for performing Mendelian randomization investigations. Wellcome Open Res 2019 4186 [Internet]. F1000 Research Limited; 2019 [cited 2021 Mar 14];4:186. Available from: https://wellcomeopenresearch.org/articles/4-186

2. Bowden J, Smith GD, Burgess S. Mendelian randomization with invalid instruments: effect estimation and bias detection through Egger regression. 2015 [cited 2021 Mar 14];44:512–25. Available from: https://pubmed.ncbi.nlm.nih.gov/26050253/

3. Bowden J, Davey Smith G, Haycock PC, Burgess S. Consistent Estimation in Mendelian Randomization with Some Invalid Instruments Using a Weighted Median Estimator. Genet Epidemiol [Internet]. Wiley-Blackwell; 2016 [cited 2021 Apr 12];40:304. Available from: /pmc/articles/PMC4849733/

4. Verbanck M, Chen CY, Neale B, Do R. Detection of widespread horizontal pleiotropy in causal relationships inferred from Mendelian randomization between complex traits and diseases. Nat Genet. Nature Publishing Group; 2018;50:693–8.

5. Rücker G, Schwarzer G, Carpenter JR, Binder H, Schumacher M. Treatment-effect estimates adjusted for small-study effects via a limit meta-analysis. Biostatistics [Internet]. Oxford Academic; 2011 [cited 2021 Mar 25];12:122–42. Available from: https://academic.oup.com/biostatistics/article/12/1/122/391113

6. Jiang L, Zheng Z, Qi T, Kemper KE, Wray NR, Visscher PM, et al. A resource-efficient tool for mixed model association analysis of large-scale data. Nat Genet 2019 5112 [Internet]. Nature Publishing Group; 2019 [cited 2021 Mar 13];51:1749–55. Available from: https://www.nature.com/articles/s41588-019-0530-8

7. Kamat MA, Blackshaw JA, Young R, Surendran P, Burgess S, Danesh J, et al. PhenoScanner V2: an expanded tool for searching human genotype–phenotype associations. Bioinformatics [Internet]. Oxford Academic; 2019 [cited 2021 Mar 13];35:4851–3. Available from: https://academic.oup.com/bioinformatics/article/35/22/4851/5522366

8. Chang CC, Chow CC, Tellier LC, Vattikuti S, Purcell SM, Lee JJ. Second-generation PLINK: Rising to the challenge of larger and richer datasets. Gigascience. Oxford Academic; 2015;4:7.

**Table S1.** Summary of lead SNPs for the genetic instrument of IEAA.

| rsID | Chr | Pos | Effect | Other | EAF  exposure | Beta  exposure | SE  exposure | P | SNP filters* | Selected for genetic instrument | Selected for conservative genetic instrument | Significant Associations with Confounders in UK Biobank | Significant Associations with Phenotypes in Phenoscanner |
| --- | --- | --- | --- | --- | --- | --- | --- | --- | --- | --- | --- | --- | --- |
| rs3917672 | 1 | 169592981 | A | G | 0.4858 | -0.2622 | 0.0304 | 5.95E-18 | Pass | Yes | Yes | - | - |
| rs7550821 | 1 | 208029947 | T | C | 0.2441 | -0.2551 | 0.035 | 3.27E-13 | Pass | Yes | No, significant associations identified by phenoscaner | - | Granulocyte count;0.02387;1.368e-08\|Myeloid white cell count;0.02552;1.388e-09\|Neutrophil count;0.02308;3.708e-08\|Sum basophil neutrophil counts;0.02355;2.072e-08\|Sum neutrophil eosinophil counts;0.02368;1.679e-08\|White blood cell count;0.02532;1.625e-09 |
| rs12043492 | 1 | 39457006 | T | C | 0.4212 | 0.2166 | 0.0313 | 4.35E-12 | Pass | Yes | Yes | - | - |
| rs6577536 | 1 | 8910110 | A | G | 0.4843 | 0.1968 | 0.0302 | 6.73E-11 | Pass | Yes | No, significant associations identified by phenoscaner | - | Basophil count;-0.01971;2.022e-08\|Granulocyte count;-0.02378;4.199e-11\|Myeloid white cell count;-0.02292;2.263e-10\|Neutrophil count;-0.02318;1.131e-10\|Plateletcrit;-0.02727;1.059e-13\|Sum basophil neutrophil counts;-0.02351;6.592e-11\|Sum neutrophil eosinophil counts;-0.02344;7.291e-11\|White blood cell count;-0.021;5.297e-09 |
| rs1726672 | 1 | 236519502 | T | C | 0.3036 | -0.2038 | 0.0329 | 6.05E-10 | Pass | Yes | Yes | - | - |
| rs2275558 | 1 | 164529120 | A | G | 0.2212 | -0.2335 | 0.0402 | 6.44E-09 | Pass | Yes | Yes | - | - |
| rs4240228 | 2 | 16688759 | T | G | 0.7168 | 0.2547 | 0.0334 | 2.47E-14 | Pass | Yes | No, significant associations identified by phenoscaner | - | Mean platelet volume;0.03759;1.631e-20 |
| rs6414374 | 3 | 150001224 | A | G | 0.1625 | 0.3214 | 0.0418 | 1.42E-14 | Pass | Yes | Yes | - | - |
| rs7627756 | 3 | 160217483 | A | G | 0.5615 | 0.2162 | 0.0301 | 6.28E-13 | Pass | Yes | Yes | - | - |
| rs2492286 | 3 | 128336298 | T | G | 0.1519 | 0.2808 | 0.0428 | 5.29E-11 | Pass | Yes | No, significant associations identified by phenoscaner | - | Eosinophil count;-0.04315;4.079e-18\|Eosinophil percentage of granulocytes;-0.04219;2.785e-17\|Eosinophil percentage of white cells;-0.04229;1.682e-17\|Neutrophil percentage of granulocytes;0.0412;1.525e-16\|Sum eosinophil basophil counts;-0.04253;1.302e-17\|Eosinophil counts;-0.04315;4e-18 |
| rs79111787 | 3 | 47715545 | T | C | 0.9678 | -0.9076 | 0.1412 | 1.28E-10 | Pass | Yes | Yes | - | - |
| rs184852236 | 3 | 47967917 | A | G | 0.0315 | 0.8613 | 0.1393 | 6.31E-10 | Pass | Yes | Yes | - | - |
| rs114933663 | 3 | 46757860 | T | C | 0.025 | 0.8732 | 0.1419 | 7.54E-10 | Pass | Yes | Yes | - | - |
| rs1488106 | 3 | 168859006 | T | C | 0.3721 | 0.1827 | 0.0311 | 4.25E-09 | Pass | Yes | No, significant associations identified by phenoscaner | - | Platelet count;0.02246;3.422e-09\|Plateletcrit;0.03555;1.127e-20\|Forced expiratory volume in 1-second, best measure;-0.01321;2.489e-08 |
| rs146179438 | 3 | 48229366 | A | C | 0.0358 | 0.757 | 0.1293 | 4.84E-09 | Pass | Yes | No, significant associations identified by phenoscaner | - | Macrophage inflammatory protein 1b levels;-0.2963;8e-28 |
| rs115744844 | 3 | 48632756 | A | C | 0.9707 | -0.7599 | 0.1312 | 7.02E-09 | Pass | Yes | Yes | - | - |
| rs2860228 | 3 | 128209667 | T | C | 0.4125 | 0.1678 | 0.0304 | 3.45E-08 | Pass | Yes | No, significant associations identified by phenoscaner | - | Basophil percentage of granulocytes;0.02157;1.192e-09\|Eosinophil count;0.02319;1.337e-10\|Eosinophil percentage of granulocytes;0.03106;9.862e-18\|Eosinophil percentage of white cells;0.02757;2.13e-14\|Lymphocyte percentage of white cells;0.02547;1.661e-12\|Myeloid white cell count;-0.02036;2.332e-08\|Neutrophil count;-0.02272;3.715e-10\|Neutrophil percentage of granulocytes;-0.03338;3.156e-20\|Neutrophil percentage of white cells;-0.02894;1.145e-15\|Sum basophil neutrophil counts;-0.02258;5.081e-10\|Sum eosinophil basophil counts;0.02446;1.311e-11 |
| rs6806687 | 3 | 128391789 | T | C | 0.3565 | -0.1724 | 0.0313 | 3.57E-08 | Pass | Yes | No, significant associations identified by phenoscaner | - | Basophil count;0.04368;4.585e-33\|Basophil percentage of granulocytes;0.04287;7.411e-32\|Basophil percentage of white cells;0.04278;2.896e-32\|Eosinophil count;0.02278;8.887e-10\|Eosinophil percentage of granulocytes;0.02105;1.648e-08\|Granulocyte percentage of myeloid white cells;-0.06125;7.55e-61\|Monocyte count;0.07179;4.988e-83\|Monocyte percentage of white cells;0.07397;1.998e-88\|Neutrophil percentage of granulocytes;-0.02805;5.429e-14\|Sum eosinophil basophil counts;0.03075;1.381e-16 |
| rs144317085 | 4 | 105806108 | A | T | 0.9612 | 0.5136 | 0.0831 | 6.42E-10 | Pass | Yes | No, significant associations identified by phenoscaner | - | Lymphocyte percentage of white cells;0.06064;1.083e-09\|Monocyte count;-0.05531;3.03e-08 |
| rs2736099 | 5 | 1287340 | A | G | 0.3538 | 0.2326 | 0.0334 | 3.47E-12 | Pass | Yes | No, significant associations identified by phenoscaner | - | Granulocyte count;-0.03019;5.63e-15\|Lymphocyte percentage of white cells;0.02129;2.805e-08\|Mean corpuscular hemoglobin;0.03569;7.511e-21\|Mean corpuscular volume;0.03425;2.038e-19\|Myeloid white cell count;-0.03027;5.593e-15\|Neutrophil count;-0.0314;3.857e-16\|Neutrophil percentage of white cells;-0.02456;1.607e-10\|Platelet count;-0.03245;1.416e-16\|Plateletcrit;-0.04821;1.907e-34\|Red blood cell count;-0.02658;3.853e-12\|Sum basophil neutrophil counts;-0.03105;9.135e-16\|Sum neutrophil eosinophil counts;-0.0304;3.311e-15\|White blood cell count;-0.02589;1.97e-11\|Intrinsic epigenetic age acceleration;-0.631;1e-12\|Serous boarderline ovarian cancer;-0.1957;3.725e-08\|Low grade and borderline serous ovarian cancer;-0.1782;1.429e-09 |
| rs10949481 | 6 | 18121029 | A | T | 0.9468 | 1.0823 | 0.0699 | 4.56E-54 | Pass | Yes | Yes | - | - |
| rs10949483 | 6 | 18122506 | A | G | 0.392 | 0.3697 | 0.0314 | 4.46E-32 | Pass | Yes | Yes | - | - |
| rs10447389 | 6 | 25642577 | A | G | 0.2687 | -0.2764 | 0.034 | 4.42E-16 | Pass | Yes | No, significant associations identified by phenoscaner | - | Hemoglobin concentration;-0.02389;1.805e-09\|Mean corpuscular hemoglobin;-0.0487;7.844e-35\|Mean corpuscular hemoglobin concentration;-0.03865;2.25e-23\|Mean corpuscular volume;-0.03425;3.795e-18\|Mean platelet volume;0.03562;1.657e-18\|Platelet count;-0.02351;8.739e-09\|Platelet distribution width;0.02701;2.058e-11\|Red cell distribution width;0.03531;5.268e-19\|Disorders of mineral metabolism;-0.0007555;3.993e-16 |
| rs116642342 | 6 | 18424879 | C | G | 0.9675 | 0.8088 | 0.103 | 4.21E-15 | Pass | Yes | Yes | - | - |
| rs9396833 | 6 | 18101320 | A | G | 0.8782 | 0.37 | 0.0587 | 2.96E-10 | Pass | Yes | Yes | - | - |
| rs6914699 | 6 | 29934022 | T | C | 0.607 | -0.1923 | 0.0325 | 3.25E-09 | Pass | Yes | No, significant associations identified by phenoscaner | - | Eosinophil count;0.0491;1.379e-40\|Eosinophil percentage of granulocytes;0.03138;1.925e-17\|Eosinophil percentage of white cells;0.03525;9.073e-22\|Granulocyte count;0.03746;5.067e-24\|High light scatter percentage of red cells;-0.04001;2.81e-27\|High light scatter reticulocyte count;-0.03884;8.69e-26\|Immature fraction of reticulocytes;-0.04021;4.921e-28\|Lymphocyte count;0.02126;1.05e-08\|Mean corpuscular hemoglobin;-0.02275;4.315e-10\|Mean corpuscular hemoglobin concentration;-0.02122;2.874e-09\|Monocyte count;0.03506;1.895e-21\|Myeloid white cell count;0.03942;2.616e-26\|Neutrophil count;0.03385;5.287e-20\|Neutrophil percentage of granulocytes;-0.02787;4.508e-14\|Platelet count;0.02193;5.419e-09\|Reticulocyte count;-0.02882;7.421e-15\|Reticulocyte fraction of red cells;-0.03073;1.058e-16\|Sum basophil neutrophil counts;0.03401;4.094e-20\|Sum eosinophil basophil counts;0.04807;6.799e-39\|Sum neutrophil eosinophil counts;0.03745;4.435e-24\|White blood cell count;0.04261;1.044e-30\|Primary sclerosing cholangitis;-0.1989;1.61e-11\|Fluid intelligence score;-0.05658;1.056e-09\|Intestinal malabsorption;-0.0006716;5.116e-10\|Self-reported malabsorption or coeliac disease;-0.002034;4.553e-35\|Self-reported multiple sclerosis;-0.001069;1.665e-12\|Rheumatoid arthritis;0.1278;9.2e-16\|Rheumatoid arthritis;0.09431;3.5e-13 |
| rs72839144 | 6 | 18011452 | T | C | 0.0917 | -0.3353 | 0.0608 | 3.55E-08 | Pass | Yes | Yes | - | - |
| rs141735437 | 6 | 17636527 | T | C | 0.0214 | -0.8468 | 0.1541 | 3.91E-08 | Pass | Yes | Yes | - | - |
| rs12666349 | 7 | 31728180 | T | C | 0.8028 | 0.2548 | 0.0436 | 5.23E-09 | Pass | Yes | Yes | - | - |
| rs10732882 | 11 | 57111693 | T | G | 0.4059 | -0.2405 | 0.0318 | 3.68E-14 | Pass | Yes | Yes | - | - |
| rs61888887 | 11 | 57099051 | T | G | 0.1146 | 0.3379 | 0.0484 | 2.96E-12 | Pass | Yes | Yes | - | - |
| rs10735418 | 12 | 107343376 | T | C | 0.6278 | 0.1954 | 0.0321 | 1.14E-09 | Pass | Yes | No, significant associations identified by phenoscaner | - | Basal metabolic rate;-0.009879;2.985e-09\|Height;-0.01367;1.892e-14\|Leg fat-free mass left;-0.009514;8.754e-09\|Leg fat-free mass right;-0.009702;4.382e-09\|Leg predicted mass left;-0.009431;9.367e-09\|Leg predicted mass right;-0.009461;8.371e-09\|Trunk fat-free mass;-0.009244;5.114e-09\|Trunk predicted mass;-0.009268;4.155e-09\|Whole body fat-free mass;-0.009619;1.39e-09\|Whole body water mass;-0.009446;2.867e-09 |
| rs12903325 | 15 | 50353277 | T | G | 0.7596 | -0.2216 | 0.0357 | 5.48E-10 | Pass | Yes | No, significant associations identified by phenoscaner | - | Basophil percentage of white cells;-0.02269;2.83e-08 |
| rs34003787 | 16 | 73071381 | T | C | 0.0862 | 0.3239 | 0.0581 | 2.54E-08 | Pass | Yes | Yes | - | - |
| rs1511762 | 18 | 42119324 | T | C | 0.2186 | 0.2642 | 0.0374 | 1.53E-12 | Pass | No, significant associations with confounders identified in UK Biobank GWAS | No, significant associations identified by phenoscaner | Systolic blood pressure (UKB_ID:4080) | Plateletcrit;-0.0295;3.291e-11 |
| rs57941717 | 21 | 38374179 | T | G | 0.2528 | 0.2908 | 0.0357 | 3.71E-16 | Pass | Yes | Yes | - | - |
| rs75243280 | 22 | 17601466 | T | C | 0.6686 | -0.2323 | 0.0339 | 7.37E-12 | Pass | Yes | Yes | - | - |

*SNP filters: filtering ambiguous and palindromic SNPs with intermediate allele frequency.

**Table S2.** Summary of lead SNPs for the genetic instrument of HannumAA

| rsID | Chr | Pos | Effect | Other | EAF  exposure | Beta  exposure | SE  exposure | P | SNP filters* | Selected for genetic instrument | Selected for conservative genetic instrument | Significant Associations with Confounders in UK Biobank | Significant Associations with Phenotypes in Phenoscanner |
| --- | --- | --- | --- | --- | --- | --- | --- | --- | --- | --- | --- | --- | --- |
| rs4383328 | 2 | 16693124 | A | T | 0.2848 | -0.1889 | 0.0329 | 9.57E-09 | Pass | Yes | No, significant associations identified by phenoscaner | - | Mean platelet volume;0.03728;3.16e-20 |
| rs1598856 | 4 | 103446115 | A | G | 0.4492 | 0.1858 | 0.0296 | 3.63E-10 | Pass | Yes | No, significant associations identified by phenoscaner | - | Lymphocyte count;0.03009;8.292e-17\|Lymphocyte percentage of white cells;0.02648;1.312e-13\|Neutrophil percentage of white cells;-0.02006;2.128e-08\|Allergic disease;-0.0322;2.293e-08\|Primary biliary cholangitis;0.2311;2e-10\|Hayfever, allergic rhinitis or eczema;-0.005699;3.427e-08 |
| rs1383732 | 4 | 103953389 | A | G | 0.8437 | 0.2287 | 0.0416 | 3.73E-08 | Pass | Yes | Yes | - | - |
| rs3093956 | 6 | 31426967 | T | C | 0.8142 | -0.2428 | 0.0396 | 8.39E-10 | Pass | No, significant associations with confounders identified in UK Biobank GWAS | No, significant associations identified by phenoscaner | Diastolic blood pressure (UKB_ID:4079); Diabetes mellitus medicatoin (UKB_ID:6177_3) | Basophil count;0.03441;1.821e-14\|Eosinophil count;0.05573;4.149e-34\|Eosinophil percentage of granulocytes;0.03326;4.37e-13\|Eosinophil percentage of white cells;0.03239;1.392e-12\|Granulocyte count;0.05133;7.893e-29\|Hematocrit;0.02891;1.755e-10\|Hemoglobin concentration;0.0311;8.293e-12\|High light scatter percentage of red cells;0.03404;1.376e-13\|High light scatter reticulocyte count;0.0391;1.916e-17\|Lymphocyte count;0.07674;5.575e-62\|Mean platelet volume;-0.02666;1.006e-08\|Monocyte count;0.04891;1.394e-26\|Myeloid white cell count;0.05367;3.219e-31\|Neutrophil count;0.04565;2.998e-23\|Neutrophil percentage of granulocytes;-0.03291;7.625e-13\|Platelet count;0.04984;1.91e-26\|Plateletcrit;0.04165;7.754e-19\|Red blood cell count;0.03154;4.525e-12\|Reticulocyte count;0.05065;4.244e-28\|Reticulocyte fraction of red cells;0.04429;6.745e-22\|Sum basophil neutrophil counts;0.0466;4.424e-24\|Sum eosinophil basophil counts;0.05864;1.668e-37\|Sum neutrophil eosinophil counts;0.05027;8.34e-28\|White blood cell count;0.07413;1.899e-58\|IgA deficiency;-1.173;1.957e-99\|Primary sclerosing cholangitis;-0.9119;1.87e-131\|Arm fat-free mass left;-0.01384;3.271e-12\|Arm fat-free mass right;-0.0137;1.963e-12\|Arm predicted mass left;-0.01433;4.492e-13\|Arm predicted mass right;-0.01315;1.212e-11\|Asthma;-0.006555;5.756e-11\|Basal metabolic rate;-0.01605;9.389e-15\|Comparative body size at age 10;-0.01302;1.165e-09\|Diastolic blood pressure;0.01882;2.972e-09\|Doctor diagnosed sarcoidosis;-0.003854;1.677e-21\|Forced expiratory volume in 1-second, predicted percentage;0.03335;8.865e-10\|Height;-0.01861;5.119e-17\|Impedance of leg left;0.0158;2.93e-08\|Impedance of leg right;0.0164;6.563e-09\|Intestinal malabsorption;-0.002955;1.035e-106\|Leg fat-free mass left;-0.01675;3.91e-16\|Leg fat-free mass right;-0.01753;1.51e-17\|Leg predicted mass left;-0.01674;2.516e-16\|Leg predicted mass right;-0.01735;2.014e-17\|Medication for cholesterol, blood pressure or diabetes: insulin;-0.003863;5.606e-13\|Medication for pain relief, constipation, heartburn: none of the above;-0.01212;9.178e-15\|Medication for pain relief, constipation, heartburn: paracetamol;0.009241;9.017e-13\|Mouth or teeth dental problems: dentures;-0.007064;1.791e-09\|Number of incorrect matches in round;0.01675;3.913e-08\|Other serious medical condition or disability diagnosed by doctor;-0.008622;1.47e-11\|Self-reported adrenocortical insufficiency or addisons disease;-0.0004765;1.043e-13\|Self-reported asthma;-0.006447;1.27e-10\|Self-reported hyperthyroidism or thyrotoxicosis;-0.004671;1.505e-66\|Self-reported hypothyroidism or myxoedema;-0.007458;5.044e-29\|Self-reported malabsorption or coeliac disease;-0.008118;0\|Self-reported sarcoidosis;-0.001257;5.937e-20\|Self-reported sjogrens syndrome or sicca syndrome;-0.0006784;4.197e-13\|Self-reported systemic lupus erythematosis or sle;-0.0006962;1.51e-11\|Self-reported type 1 diabetes;-0.0005447;2.389e-09\|Sitting height;-0.02706;3.758e-29\|Started insulin within one year diagnosis of diabetes;-0.04799;1.829e-24\|Treatment with carbimazole;-0.0005579;4.521e-10\|Treatment with fludrocortisone;-0.0004543;4.535e-12\|Treatment with insulin;-0.002834;3.762e-14\|Treatment with insulin product;-0.003292;1.9e-26\|Treatment with levothyroxine sodium;-0.007115;5.503e-31\|Treatment with paracetamol;0.007602;3.071e-10\|Treatment with thyroxine product;-0.002502;8.016e-14\|Trunk fat-free mass;-0.01476;6.38e-14\|Trunk predicted mass;-0.01466;7.65e-14\|Unspecified haematuria;0.003293;6.322e-13\|Weight;-0.01719;3.032e-10\|Whole body fat-free mass;-0.01596;6.585e-16\|Whole body water mass;-0.01588;9.931e-16\|Schizophrenia;0.1268;8.958e-16 |
| rs3130985 | 6 | 31085356 | T | C | 0.1535 | 0.2354 | 0.0421 | 2.20E-08 | Pass | No, significant associations with confounders identified in UK Biobank GWAS | No, significant associations identified by phenoscaner | Diabetes diagnosed by doctor (UKB_ID:2443); Diastolic blood pressure (UKB_ID:4079); Diabetes mellitus medicatoin (UKB_ID:6177_3) | Basophil count;0.04104;2.248e-17\|Eosinophil count;0.0604;1.814e-34\|Eosinophil percentage of granulocytes;0.03242;5.709e-11\|Eosinophil percentage of white cells;0.03063;5.131e-10\|Granulocyte count;0.06444;1.658e-38\|Hematocrit;0.0361;1.434e-13\|Hemoglobin concentration;0.04014;2.772e-16\|High light scatter percentage of red cells;0.03395;7.538e-12\|High light scatter reticulocyte count;0.03973;1.127e-15\|Lymphocyte count;0.09484;6.694e-81\|Monocyte count;0.07264;5.958e-49\|Myeloid white cell count;0.06883;1.774e-43\|Neutrophil count;0.05825;6.178e-32\|Neutrophil percentage of granulocytes;-0.03255;4.777e-11\|Platelet count;0.04129;2.778e-16\|Plateletcrit;0.03545;2.469e-12\|Red blood cell count;0.03659;9.465e-14\|Red cell distribution width;-0.02766;1.625e-08\|Reticulocyte count;0.05549;5.791e-29\|Reticulocyte fraction of red cells;0.04788;5.227e-22\|Sum basophil neutrophil counts;0.05945;4.439e-33\|Sum eosinophil basophil counts;0.06489;1.958e-39\|Sum neutrophil eosinophil counts;0.06319;3.27e-37\|White blood cell count;0.09417;1.874e-80\|IgA deficiency;-1.198;1.14e-84\|Primary sclerosing cholangitis;-1.102;1.404e-222\|Arm fat-free mass left;-0.01834;7.41e-18\|Arm fat-free mass right;-0.01806;5.198e-18\|Arm predicted mass left;-0.01854;2.395e-18\|Arm predicted mass right;-0.01727;1.033e-16\|Asthma;-0.00867;6.735e-16\|Basal metabolic rate;-0.02139;6.164e-22\|Comparative body size at age 10;-0.01673;3.149e-13\|Comparative height size at age 10;-0.01307;1.115e-08\|Diabetes diagnosed by doctor;-0.004003;2.514e-08\|Diastolic blood pressure;0.02187;1.288e-10\|Doctor diagnosed sarcoidosis;-0.004282;7.383e-23\|Forced expiratory volume in 1-second, predicted percentage;0.04012;5.815e-12\|Height;-0.02823;1.964e-32\|Hip circumference;-0.0229;5.168e-12\|Impedance of leg right;0.01692;2.395e-08\|Intestinal malabsorption;-0.003364;5.562e-120\|Leg fat-free mass left;-0.02184;4.168e-23\|Leg fat-free mass right;-0.0227;7.553e-25\|Leg predicted mass left;-0.02169;4.2e-23\|Leg predicted mass right;-0.0224;1.564e-24\|Long-standing illness, disability or infirmity;-0.009098;1.031e-08\|Medication for cholesterol, blood pressure or diabetes: insulin;-0.004367;3.148e-14\|Medication for pain relief, constipation, heartburn: none of the above;-0.01297;1.096e-14\|Medication for pain relief, constipation, heartburn: paracetamol;0.009764;1.952e-12\|Mouth or teeth dental problems: dentures;-0.008074;1.469e-10\|Number of days or week of moderate physical activity 10+ minutes;0.04499;2.037e-08\|Other serious medical condition or disability diagnosed by doctor;-0.009966;3.495e-13\|Self-reported adrenocortical insufficiency or addisons disease;-0.0004477;7.444e-11\|Self-reported asthma;-0.008621;1.096e-15\|Self-reported hyperthyroidism or thyrotoxicosis;-0.005228;2.699e-72\|Self-reported hypothyroidism or myxoedema;-0.007825;7.862e-28\|Self-reported malabsorption or coeliac disease;-0.00904;0\|Self-reported psoriasis;0.00285;1.758e-15\|Self-reported sarcoidosis;-0.001425;4.494e-22\|Self-reported sjogrens syndrome or sicca syndrome;-0.0007289;3.865e-13\|Self-reported systemic lupus erythematosis or sle;-0.0008928;7.339e-16\|Self-reported type 1 diabetes;-0.0006159;3.149e-10\|Sitting height;-0.03678;8.837e-46\|Started insulin within one year diagnosis of diabetes;-0.05372;6.465e-27\|Treatment with carbimazole;-0.000596;5.346e-10\|Treatment with fludrocortisone;-0.0004157;3.588e-09\|Treatment with insulin;-0.002963;1.561e-13\|Treatment with insulin product;-0.00368;1.391e-28\|Treatment with levothyroxine sodium;-0.007747;7.217e-32\|Treatment with thyroxine product;-0.002672;1.045e-13\|Trunk fat-free mass;-0.01976;7.956e-21\|Trunk predicted mass;-0.01961;1.17e-20\|Unspecified haematuria;0.003958;7.659e-16\|Weight;-0.02354;8.981e-16\|Whole body fat-free mass;-0.02092;5.455e-23\|Whole body water mass;-0.02085;8.647e-23\|Schizophrenia;0.1524;8.729e-19 |
| rs111731678 | 7 | 130418744 | A | T | 0.1939 | -0.2266 | 0.0398 | 1.26E-08 | Pass | Yes | Yes | - | - |
| rs10786282 | 10 | 98122808 | A | G | 0.2124 | -0.36 | 0.0362 | 2.61E-23 | Pass | Yes | Yes | - | - |
| rs1005277 | 10 | 38218259 | A | C | 0.2982 | 0.3006 | 0.033 | 8.92E-20 | Pass | Yes | Yes | - | Extrinsic epigenetic age acceleration;0.7366;1e-13 |
| rs117932856 | 10 | 38164764 | A | T | 0.04 | 0.7878 | 0.0912 | 5.60E-18 | Pass | Yes | Yes | - | - |
| rs144646268 | 10 | 98062753 | T | G | 0.0439 | -0.6295 | 0.0743 | 2.36E-17 | Pass | Yes | Yes | - | - |
| rs150082435 | 10 | 38549439 | A | G | 0.0531 | 0.6264 | 0.0823 | 2.79E-14 | Pass | Yes | Yes | - | - |
| rs1831315 | 10 | 38473353 | T | C | 0.5942 | -0.23 | 0.0309 | 1.06E-13 | Pass | Yes | Yes | - | - |
| rs10764140 | 10 | 37909295 | T | G | 0.746 | -0.2409 | 0.0348 | 4.50E-12 | Pass | Yes | Yes | - | - |
| rs75666621 | 10 | 37709477 | T | C | 0.9578 | -0.5337 | 0.0785 | 1.03E-11 | Pass | Yes | Yes | - | - |
| rs2804650 | 10 | 38739704 | A | G | 0.4435 | 0.2004 | 0.0313 | 1.56E-10 | Pass | Yes | Yes | - | - |
| rs117267867 | 10 | 42939635 | T | C | 0.0277 | 0.7126 | 0.1135 | 3.36E-10 | Pass | Yes | Yes | - | - |
| rs10882762 | 10 | 98007727 | A | G | 0.4219 | -0.1827 | 0.0309 | 3.52E-09 | Pass | Yes | Yes | - | - |
| rs11146769 | 10 | 39110470 | T | C | 0.4419 | 0.2018 | 0.0346 | 5.51E-09 | Pass | Yes | Yes | - | - |
| rs4838595 | 10 | 49675247 | T | C | 0.1217 | -0.2576 | 0.0453 | 1.29E-08 | Pass | Yes | Yes | - | - |
| rs1208784 | 10 | 37768433 | A | G | 0.4586 | -0.1688 | 0.0302 | 2.40E-08 | Pass | Yes | Yes | - | - |
| rs117065021 | 10 | 37249141 | T | G | 0.0295 | 0.5625 | 0.1028 | 4.53E-08 | Pass | Yes | Yes | - | - |
| rs72829536 | 10 | 98167265 | T | C | 0.1637 | -0.2233 | 0.0409 | 4.80E-08 | Pass | Yes | Yes | - | - |
| rs12417758 | 11 | 66076360 | T | C | 0.5461 | -0.2093 | 0.0304 | 6.22E-12 | Pass | Yes | Yes | - | - |
| rs34970912 | 16 | 73068163 | C | G | 0.9648 | -0.5213 | 0.0939 | 2.88E-08 | Pass | Yes | No, significant associations identified by phenoscaner | - | Height;-0.02881;2.002e-09 |

*SNP filters: filtering ambiguous and palindromic SNPs with intermediate allele frequency.

**Table S3.** Summary of lead SNPs for the genetic instrument of GrimAA

| rsID | Chr | Pos | Effect | Other | EAF  exposure | Beta  exposure | SE  exposure | P | SNP filters* | Selected for genetic instrument | Selected for conservative genetic instrument | Significant Associations with Confounders in UK Biobank | Significant Associations with Phenotypes in Phenoscanner |
| --- | --- | --- | --- | --- | --- | --- | --- | --- | --- | --- | --- | --- | --- |
| rs9386796 | 6 | 109618704 | T | C | 0.4597 | 0.1983 | 0.0294 | 1.64E-11 | Pass | Yes | Yes. One of the most significant three SNPs associated with GrimAA despite associations found in Phenoscanner | - | Basophil count;-0.03192;1.09e-19\|Basophil percentage of white cells;-0.02285;5.728e-11\|Granulocyte count;-0.03309;4.496e-20\|High light scatter percentage of red cells;-0.03081;1.179e-17\|High light scatter reticulocyte count;-0.02165;1.842e-09\|Immature fraction of reticulocytes;-0.02052;8.757e-09\|Lymphocyte percentage of white cells;0.02879;8.559e-16\|Mean corpuscular hemoglobin;-0.08786;1.94e-135\|Mean corpuscular hemoglobin concentration;-0.04106;3.911e-32\|Mean corpuscular volume;-0.08116;1.34e-116\|Myeloid white cell count;-0.03331;3.172e-20\|Neutrophil count;-0.03167;1.344e-18\|Neutrophil percentage of white cells;-0.02582;5.875e-13\|Platelet count;-0.02799;2.069e-14\|Plateletcrit;-0.02568;2.625e-12\|Red blood cell count;0.06019;7.152e-64\|Red cell distribution width;0.05989;1.044e-63\|Reticulocyte fraction of red cells;-0.03194;8.141e-19\|Sum basophil neutrophil counts;-0.03241;2.366e-19\|Sum neutrophil eosinophil counts;-0.03236;2.561e-19\|White blood cell count;-0.02698;6.743e-14\|Mean corpuscular hemoglobin concentration;NA;2.85e-23\|Mean corpuscular volume;NA;1.3e-21\|Red blood cell count RBC;NA;2.27e-17 |
| rs887466 | 6 | 31143511 | A | G | 0.3815 | -0.1928 | 0.031 | 5.09E-10 | Pass | Yes | Yes. One of the most significant three SNPs associated with GrimAA despite associations found in Phenoscanner | - | Eosinophil percentage of granulocytes;-0.02022;4.842e-08\|Eosinophil percentage of white cells;-0.02228;1.564e-09\|Hematocrit;-0.02625;6.897e-13\|Hemoglobin concentration;-0.02715;1.443e-13\|High light scatter percentage of red cells;-0.0204;3.937e-08\|High light scatter reticulocyte count;-0.0248;2.412e-11\|Red blood cell count;-0.02773;4.712e-14\|Reticulocyte count;-0.0313;3.977e-17\|Reticulocyte fraction of red cells;-0.02531;9.948e-12\|IgA deficiency;0.5307;3.307e-24\|IgA deficiency;0.59;9.43e-09\|Age-related macular degeneration;NA;2.1e-08\|Primary sclerosing cholangitis;0.388;5.982e-38\|Ankylosing spondylitis;-0.0002833;2.758e-09\|Arm fat-free mass left;0.01196;9.91e-14\|Arm fat-free mass right;0.01185;5.499e-14\|Arm predicted mass left;0.01189;1.12e-13\|Arm predicted mass right;0.01139;4.026e-13\|Basal metabolic rate;0.01411;3.797e-17\|Height;0.01453;6.087e-16\|Hip circumference;0.01713;7.833e-12\|Impedance of leg left;-0.01571;9.341e-12\|Impedance of leg right;-0.01409;7.27e-10\|Intestinal malabsorption;0.0006452;3.293e-09\|Leg fat-free mass left;0.01462;1.582e-18\|Leg fat-free mass right;0.01448;3.244e-18\|Leg predicted mass left;0.01453;1.496e-18\|Leg predicted mass right;0.01443;2.597e-18\|Self-reported ankylosing spondylitis;-0.001896;2.524e-44\|Self-reported enlarged prostate;-0.00171;4.909e-09\|Self-reported hyperthyroidism or thyrotoxicosis;0.001288;4.367e-09\|Self-reported hypothyroidism or myxoedema;0.007654;1.301e-45\|Self-reported malabsorption or coeliac disease;0.001726;2.764e-25\|Self-reported multiple sclerosis;0.001008;4.232e-11\|Self-reported psoriasis;0.002683;3.122e-23\|Sitting height;0.01736;6.633e-19\|Treatment with levothyroxine sodium;0.006365;1.865e-37\|Treatment with thyroxine product;0.001812;2.377e-11\|Trunk fat-free mass;0.01364;1.105e-17\|Trunk predicted mass;0.01366;7.799e-18\|Weight;0.01586;7.071e-13\|Whole body fat-free mass;0.01395;2.597e-18\|Whole body water mass;0.014;2.304e-18\|Rheumatoid arthritis;-0.09531;8e-13 |
| rs3134745 | 6 | 31242762 | T | C | 0.3016 | 0.185 | 0.0333 | 2.87E-08 | Pass | No, significant associations with confounders identified in UK Biobank GWAS | No, significant associations identified by phenoscaner | Diabetes mellitus medicatoin (UKB_ID:6177_3) | Hematocrit;-0.0329;2.066e-18\|Hemoglobin concentration;-0.04099;1.816e-27\|High light scatter percentage of red cells;-0.03452;1.462e-19\|High light scatter reticulocyte count;-0.04083;1.031e-26\|Mean corpuscular hemoglobin concentration;-0.02409;6.375e-11\|Mean corpuscular volume;0.02089;2.498e-08\|Mean platelet volume;0.03779;1.146e-22\|Platelet count;-0.03692;1.942e-21\|Plateletcrit;-0.02207;1.448e-08\|Red blood cell count;-0.03986;5.461e-26\|Red cell distribution width;0.0323;1.017e-17\|Reticulocyte count;-0.0511;9.56e-41\|Reticulocyte fraction of red cells;-0.04267;5.86e-29\|IgA deficiency;0.812;4.494e-64\|HIV 1 control;NA;2.6e-13\|Primary sclerosing cholangitis;0.5761;6.182e-86\|Arm predicted mass left;0.009136;2.665e-08\|Asthma;0.007025;2.759e-17\|Basal metabolic rate;0.01002;5.604e-09\|Height;0.01341;3.334e-13\|Intestinal malabsorption;0.001717;3.388e-53\|Leg fat-free mass left;0.01044;9.72e-10\|Leg fat-free mass right;0.01036;1.269e-09\|Leg predicted mass left;0.01035;1.061e-09\|Leg predicted mass right;0.01029;1.293e-09\|Medication for cholesterol, blood pressure or diabetes: insulin;0.002692;1.641e-09\|Self-reported ankylosing spondylitis;-0.000795;1.123e-08\|Self-reported asthma;0.007119;1.188e-17\|Self-reported enlarged prostate;-0.001707;1.22e-08\|Self-reported hyperthyroidism or thyrotoxicosis;0.001969;2.153e-18\|Self-reported hypothyroidism or myxoedema;0.01087;7.339e-86\|Self-reported malabsorption or coeliac disease;0.004524;1.539e-155\|Self-reported psoriasis;-0.003555;1.131e-37\|Self-reported sarcoidosis;0.0006327;2.962e-08\|Self-reported type 1 diabetes;0.0004675;6.751e-10\|Sitting height;0.01833;5.991e-20\|Started insulin within one year diagnosis of diabetes;0.03373;1.444e-17\|Treatment with insulin;0.00206;2.918e-11\|Treatment with insulin product;0.002396;1.07e-20\|Treatment with levothyroxine sodium;0.009231;3.653e-73\|Treatment with thyroxine product;0.002491;3.279e-19\|Trunk fat-free mass;0.01024;3.599e-10\|Trunk predicted mass;0.01017;4.265e-10\|Whole body fat-free mass;0.01021;4.846e-10\|Whole body water mass;0.01012;7.107e-10\|Rheumatoid arthritis;0.1044;3.3e-09 |
| rs17094148 | 10 | 101280279 | A | G | 0.7067 | -0.18 | 0.0323 | 2.55E-08 | Pass | Yes | No, significant associations identified by phenoscaner | - | Granulocyte percentage of myeloid white cells;-0.03115;3.575e-15\|Mean corpuscular hemoglobin;-0.02748;2.489e-12\|Mean corpuscular volume;-0.02653;1.177e-11\|Monocyte count;0.04666;4.597e-32\|Monocyte percentage of white cells;0.03831;2.985e-22\|Plateletcrit;0.02526;4.613e-10\|Crohns disease;0.1625;5.564e-10\|Inflammatory bowel disease;0.1544;4.652e-16\|Ulcerative colitis;0.1523;1.999e-10 |
| rs4065321 | 17 | 38143548 | T | C | 0.5333 | -0.1703 | 0.0296 | 8.78E-09 | Pass | Yes | Y. One of the most significant three SNPs associated with GrimAA despite associations found in Phenoscanner | - | Basophil count;0.05073;2.043e-47\|Eosinophil percentage of granulocytes;-0.03298;3.755e-20\|Granulocyte count;0.1143;1.64e-221\|Granulocyte percentage of myeloid white cells;0.07979;4.38e-110\|Lymphocyte percentage of white cells;-0.07833;1.04e-106\|Monocyte count;0.02235;4.275e-10\|Monocyte percentage of white cells;-0.05902;2.167e-61\|Myeloid white cell count;0.1114;1.55e-209\|Neutrophil count;0.1134;4.34e-219\|Neutrophil percentage of granulocytes;0.03042;2.258e-17\|Neutrophil percentage of white cells;0.08591;1.43e-127\|Sum basophil neutrophil counts;0.1142;1.54e-221\|Sum eosinophil basophil counts;0.02731;2.323e-14\|Sum neutrophil eosinophil counts;0.1135;2.92e-219\|White blood cell count;0.09842;2.67e-165\|Allergic disease;0.0322;1.84e-08\|White blood cell count;NA;2.94e-14\|White blood cell count;NA;1.43e-12\|White blood cell count;-0.094;3e-14\|White blood cell count;0.14;1e-12\|White blood cell count basophil;-0.05073;2e-47\|Asthma;0.005218;2.164e-11\|No blood clot, bronchitis, emphysema, asthma, rhinitis, eczema or allergy diagnosed by doctor;-0.007153;3.467e-10\|Self-reported asthma;0.005328;8.923e-12\|Asthma;0.08514;2.17e-11\|Asthma;0.07993;1.75e-11 |

*SNP filters: filtering ambiguous and palindromic SNPs with intermediate allele frequency

**Table S4.** Summary of lead SNPs for the genetic instrument of PhenoAA

| rsID | Chr | Pos | Effect | Other | EAF  exposure | Beta  exposure | SE  exposure | P | SNP filters* | Selected for genetic instrument | Selected for conservative genetic instrument | Significant Associations with Confounders in UK Biobank | Significant Associations with Phenotypes in Phenoscanner |
| --- | --- | --- | --- | --- | --- | --- | --- | --- | --- | --- | --- | --- | --- |
| rs678553 | 1 | 236525447 | T | C | 0.6892 | 0.3265 | 0.0412 | 2.27E-15 | Pass | Yes | Yes | - | - |
| rs752223 | 1 | 60433076 | A | G | 0.0818 | -0.5602 | 0.072 | 7.20E-15 | Pass | Yes | Yes | - | - |
| rs6531114 | 2 | 16617781 | T | C | 0.2633 | -0.2542 | 0.0424 | 2.07E-09 | Pass | Yes | No, significant associations identified by phenoscaner | - | Mean platelet volume;-0.03994;5.844e-22 |
| rs1142345 | 6 | 18130918 | T | C | 0.9454 | 0.8235 | 0.0871 | 3.35E-21 | Pass | Yes | Yes. One of the most significant three SNPs associated with PhenoAA despite associations found in Phenoscanner | - | Cardiovascular disease prevalence;NA;4.5e-21\|Systolic blood pressure;NA;7.4e-14\|Thiopurine methyltransferase activity in acute lymphoblastic leukemia patients treated with mercaptopurines;NA;9e-61 |
| rs4294009 | 6 | 18106076 | T | G | 0.6117 | -0.3359 | 0.0393 | 1.24E-17 | Pass | Yes | Yes | - | - |
| rs1990053 | 7 | 44925896 | A | G | 0.4231 | 0.2573 | 0.0384 | 2.05E-11 | Pass | Yes | No, significant associations identified by phenoscaner | - | Mean corpuscular hemoglobin;0.03737;1.658e-25\|Mean corpuscular hemoglobin concentration;0.0196;2.225e-08\|Mean corpuscular volume;0.03197;3.456e-19\|Mean platelet volume;-0.02352;1.24e-10\|Platelet count;-0.0361;1.261e-22\|Plateletcrit;-0.05498;6.303e-50\|Red blood cell count;-0.0286;1.843e-15 |
| rs11190127 | 10 | 101271982 | A | C | 0.3786 | 0.2484 | 0.0397 | 3.83E-10 | Pass | Yes | No, significant associations identified by phenoscaner | - | Granulocyte percentage of myeloid white cells;0.03013;6.783e-16\|Mean corpuscular hemoglobin;0.0231;4.221e-10\|Mean corpuscular volume;0.02201;2.386e-09\|Monocyte count;-0.04177;4.31e-29\|Monocyte percentage of white cells;-0.03469;1.166e-20\|Plateletcrit;-0.0223;5.365e-09\|Crohns disease;-0.1707;8.537e-12\|Inflammatory bowel disease;-0.1559;7.745e-18\|Ulcerative colitis;-0.1535;1.653e-11 |
| rs11253338 | 10 | 759559 | T | C | 0.184 | 0.2846 | 0.0494 | 8.49E-09 | Pass | Yes | Yes | - | - |
| rs73028070 | 11 | 122681835 | A | G | 0.0786 | -0.4329 | 0.0768 | 1.74E-08 | Pass | Yes | Yes | - | - |
| rs3829957 | 17 | 3378876 | T | C | 0.1974 | -0.3796 | 0.0482 | 3.51E-15 | Pass | Yes | Yes | - | - |
| rs116853700 | 17 | 55466295 | A | G | 0.0425 | 0.5517 | 0.0976 | 1.59E-08 | Pass | Yes | No, significant associations identified by phenoscaner | - | Mean platelet volume;-0.0549;5.519e-10\|Platelet count;-0.04918;3.673e-08\|Plateletcrit;-0.0888;3.698e-23 |
| rs7228835 | 18 | 41969071 | C | G | 0.1217 | -0.5144 | 0.061 | 3.30E-17 | Pass | Yes | Yes. One of the most significant three SNPs associated with PhenoAA despite associations found in Phenoscanner | - | Lymphocyte percentage of white cells;-0.03217;1.606e-08\|Mean platelet volume;0.03643;3.219e-10\|Neutrophil count;0.03156;3.533e-08\|Platelet count;0.04178;8.36e-13\|Plateletcrit;0.06738;1.315e-30 |
| rs10853522 | 18 | 42240598 | A | G | 0.3728 | -0.2288 | 0.039 | 4.63E-09 | Pass | Yes | Yes | - | - |
| rs1849209 | 18 | 42161643 | T | G | 0.7587 | -0.244 | 0.0443 | 3.69E-08 | Pass | No, significant associations with confounders identified in UK Biobank GWAS | No, significant associations identified by phenoscaner | Systolic blood pressure (UKB_ID:4080) | Plateletcrit;0.0324;6.203e-14 |

*SNP filters: filtering ambiguous and palindromic SNPs with intermediate allele frequency.

**Table S5.** Summary of lead SNPs for the genetic instrument of kidney function based on serum creatinine eGFR refined by serum cystatin eGFR

| rsID | Chr | Pos | Effect | Other | EAF exposure | Beta exposure | SE exposure | P | SNP filters* | Selected for genetic instrument | Selected for conservative genetic instrument | Significant associations with confounders in UK Biobank |
| --- | --- | --- | --- | --- | --- | --- | --- | --- | --- | --- | --- | --- |
| rs267738 | 1 | 150940625 | T | G | 0.79 | -0.005007 | 0.0004211 | 1.33E-32 | Pass | Yes | Yes | - |
| rs6667182 | 1 | 15914545 | T | C | 0.32 | -0.004284 | 0.00043 | 2.22E-23 | Pass | Yes | Yes | - |
| rs1887252 | 1 | 82957871 | C | G | 0.64 | -0.002889 | 0.0003584 | 7.45E-16 | Pass | Yes | Yes | - |
| rs499600 | 1 | 46039077 | T | G | 0.15 | -0.003703 | 0.0004776 | 8.93E-15 | Pass | Yes | Yes | - |
| rs78444298 | 1 | 184672098 | A | G | 0.019 | -0.010669 | 0.0014001 | 2.53E-14 | Pass | Yes | Yes | - |
| rs2490391 | 1 | 243469669 | A | C | 0.46 | -0.002497 | 0.0003461 | 5.46E-13 | Pass | Yes | Yes | - |
| rs509345 | 1 | 150276022 | A | G | 0.52 | 0.002443 | 0.0003446 | 1.35E-12 | Pass | Yes | Yes | - |
| rs11211257 | 1 | 46581933 | A | G | 0.9 | 0.003931 | 0.0005866 | 2.07E-11 | Pass | Yes | Yes | - |
| rs7543734 | 1 | 94050911 | C | G | 0.2 | 0.00312 | 0.000482 | 9.58E-11 | Pass | Yes | Yes | - |
| rs7514450 | 1 | 220991171 | T | C | 0.43 | 0.002222 | 0.0003467 | 1.48E-10 | Pass | Yes | Yes | - |
| rs2792796 | 1 | 56715908 | T | C | 0.61 | -0.002084 | 0.0003518 | 3.14E-09 | Pass | Yes | Yes | - |
| rs11166440 | 1 | 100808363 | A | G | 0.63 | 0.002051 | 0.0003588 | 1.10E-08 | Pass | Yes | Yes | - |
| rs3795503 | 1 | 180905694 | T | C | 0.33 | 0.002156 | 0.0003808 | 1.51E-08 | Pass | Yes | Yes | - |
| rs1119066 | 1 | 186658212 | A | C | 0.15 | 0.002706 | 0.0004819 | 1.96E-08 | Pass | Yes | Yes | - |
| rs688540 | 1 | 48002447 | A | G | 0.87 | -0.003111 | 0.0005567 | 2.30E-08 | Pass | Yes | Yes | - |
| rs3845534 | 1 | 163738950 | A | G | 0.49 | -0.001903 | 0.0003463 | 3.91E-08 | Pass | Yes | Yes | - |
| rs12024377 | 1 | 205537858 | A | G | 0.37 | 0.00201 | 0.0003667 | 4.25E-08 | Pass | Yes | Yes | - |
| rs7535253 | 1 | 214744893 | T | C | 0.21 | 0.002307 | 0.0004227 | 4.86E-08 | Pass | Yes | Yes | - |
| rs6546869 | 2 | 73895765 | A | G | 0.22 | 0.006087 | 0.0004159 | 1.66E-48 | Pass | Yes | Yes | - |
| rs780094 | 2 | 27741237 | T | C | 0.38 | 0.004601 | 0.0003565 | 4.16E-38 | Pass | Yes | No, significant associations with confounders identified in UK Biobank GWAS | Cholesterol-lowering medication (UKB_ID:6153_1); Cholesterol-lowering medication (UKB_ID:6177_1) |
| rs807624 | 2 | 15782471 | T | G | 0.34 | 0.003376 | 0.0003633 | 1.53E-20 | Pass | Yes | Yes | - |
| rs35472707 | 2 | 169995581 | T | C | 0.05 | -0.007542 | 0.0008293 | 9.53E-20 | Pass | Yes | Yes | - |
| rs187355703 | 2 | 176993583 | C | G | 0.97 | 0.010107 | 0.0011431 | 9.45E-19 | Pass | Yes | Yes | - |
| rs4567937 | 2 | 18676265 | A | G | 0.32 | -0.003186 | 0.0003694 | 6.40E-18 | Pass | Yes | Yes | - |
| rs11694902 | 2 | 121988884 | A | G | 0.14 | 0.004132 | 0.000503 | 2.14E-16 | Pass | Yes | Yes | - |
| rs168505 | 2 | 54920968 | T | C | 0.4 | -0.002675 | 0.0003502 | 2.18E-14 | Pass | Yes | Yes | - |
| rs10865189 | 2 | 43433257 | C | G | 0.47 | 0.002534 | 0.0003555 | 1.02E-12 | Remove. Palindromic SNP with intermediate allele frequency | NA | NA | - |
| rs140179699 | 2 | 120936492 | A | G | 0.95 | 0.007306 | 0.0010769 | 1.16E-11 | Pass | Yes | Yes | - |
| rs12989250 | 2 | 148776438 | A | G | 0.31 | -0.00256 | 0.0003777 | 1.23E-11 | Pass | Yes | Yes | - |
| rs72995641 | 2 | 103166325 | A | G | 0.2 | -0.002592 | 0.0004279 | 1.39E-09 | Pass | Yes | Yes | - |
| rs13029395 | 2 | 227344207 | T | C | 0.18 | 0.003432 | 0.0005675 | 1.48E-09 | Remove. Not found in outcome dataset. | NA | NA | - |
| rs3791221 | 2 | 226933 | A | G | 0.65 | 0.002138 | 0.0003604 | 3.00E-09 | Pass | Yes | Yes | - |
| rs17462630 | 2 | 219286541 | C | G | 0.34 | 0.002406 | 0.0004097 | 4.32E-09 | Pass | Yes | Yes | - |
| rs7565830 | 2 | 159810691 | A | G | 0.72 | -0.002225 | 0.0003867 | 8.77E-09 | Pass | Yes | Yes | - |
| rs1595810 | 2 | 12115479 | A | G | 0.2 | -0.002371 | 0.0004332 | 4.40E-08 | Pass | Yes | Yes | - |
| rs1397764 | 3 | 141750810 | A | G | 0.28 | 0.004656 | 0.000384 | 7.83E-34 | Pass | Yes | Yes | - |
| rs112545201 | 3 | 185803532 | T | C | 0.13 | -0.004215 | 0.0005066 | 8.79E-17 | Pass | Yes | No, significant associations with confounders identified in UK Biobank GWAS | Body mass index (UKB_ID:21001) |
| rs9868185 | 3 | 121657593 | A | G | 0.54 | 0.002653 | 0.0003451 | 1.50E-14 | Pass | Yes | Yes | - |
| rs7651407 | 3 | 48443816 | T | C | 0.45 | 0.002651 | 0.0003932 | 1.56E-11 | Pass | Yes | Yes | - |
| rs3905668 | 3 | 135931586 | A | G | 0.72 | -0.00255 | 0.000383 | 2.81E-11 | Pass | Yes | No, significant associations with confounders identified in UK Biobank GWAS | Body mass index (UKB_ID:21001) |
| rs11919484 | 3 | 186432839 | T | G | 0.31 | -0.002321 | 0.0003711 | 3.98E-10 | Pass | Yes | Yes | - |
| rs4625 | 3 | 49572140 | A | G | 0.68 | -0.002324 | 0.0003724 | 4.34E-10 | Pass | Yes | No, significant associations with confounders identified in UK Biobank GWAS | Body mass index (UKB_ID:21001) |
| rs9828976 | 3 | 136536835 | C | G | 0.75 | -0.002385 | 0.0003978 | 2.04E-09 | Pass | Yes | No, significant associations with confounders identified in UK Biobank GWAS | Body mass index (UKB_ID:21001) |
| rs62257555 | 3 | 51593113 | A | G | 0.94 | 0.004827 | 0.0008671 | 2.60E-08 | Pass | Yes | Yes | - |
| rs62257807 | 3 | 50929873 | T | C | 0.06 | -0.004639 | 0.0008364 | 2.92E-08 | Pass | Yes | No, significant associations with confounders identified in UK Biobank GWAS | Body mass index (UKB_ID:21001) |
| rs28817415 | 4 | 77401452 | T | C | 0.44 | -0.007437 | 0.0003438 | 9.68E-104 | Remove. Not found in the outcome dataset. | NA | NA | - |
| rs1458038 | 4 | 81164723 | T | C | 0.3 | 0.003197 | 0.0003795 | 3.60E-17 | Pass | Yes | No, significant associations with confounders identified in UK Biobank GWAS | Cholesterol-lowering medication (UKB_ID:6177_1) |
| rs223308 | 4 | 103812499 | A | G | 0.52 | -0.002702 | 0.0003425 | 3.00E-15 | Pass | Yes | Yes | - |
| rs3812036 | 5 | 176813404 | T | C | 0.26 | -0.006869 | 0.0004059 | 3.19E-64 | Pass | Yes | Yes | - |
| rs11951093 | 5 | 39421736 | A | G | 0.42 | -0.005581 | 0.0003593 | 2.05E-54 | Pass | Yes | Yes | - |
| rs55938024 | 5 | 67742038 | A | G | 0.12 | -0.006457 | 0.0006051 | 1.37E-26 | Pass | Yes | Yes | - |
| rs79760705 | 5 | 53298716 | T | G | 0.11 | 0.005609 | 0.0005512 | 2.55E-24 | Pass | Yes | Yes | - |
| rs495237 | 5 | 39950266 | T | G | 0.25 | 0.002861 | 0.0003967 | 5.47E-13 | Pass | Yes | Yes | - |
| rs13157326 | 5 | 34504277 | A | G | 0.48 | -0.002706 | 0.0003877 | 2.95E-12 | Pass | Yes | Yes | - |
| rs6555317 | 5 | 498235 | A | G | 0.69 | 0.002385 | 0.0004108 | 6.45E-09 | Pass | Yes | Yes | - |
| rs881858 | 6 | 43806609 | A | G | 0.7 | -0.005595 | 0.0003776 | 1.15E-49 | Pass | Yes | Yes | - |
| rs6921580 | 6 | 7203714 | C | G | 0.41 | 0.002735 | 0.0003562 | 1.61E-14 | Pass | Yes | Yes | - |
| rs1268176 | 6 | 109018046 | A | G | 0.34 | 0.002728 | 0.0003643 | 7.05E-14 | Pass | Yes | Yes | - |
| rs3134605 | 6 | 32159956 | T | C | 0.8 | 0.003285 | 0.0004497 | 2.77E-13 | Pass | Yes | No, significant associations with confounders identified in UK Biobank GWAS | Body mass index (UKB_ID:21001) |
| rs9375694 | 6 | 130356608 | A | G | 0.7 | 0.002596 | 0.0003753 | 4.63E-12 | Pass | Yes | Yes | - |
| rs13200335 | 6 | 41690823 | A | C | 0.42 | 0.002361 | 0.0003498 | 1.50E-11 | Pass | Yes | Yes | - |
| rs6458868 | 6 | 52630153 | T | C | 0.65 | -0.002128 | 0.0003602 | 3.50E-09 | Pass | Yes | Yes | - |
| rs62432759 | 6 | 154858365 | A | G | 0.78 | -0.002494 | 0.0004317 | 7.56E-09 | Pass | Yes | Yes | - |
| rs10224002 | 7 | 151415041 | A | G | 0.72 | 0.006845 | 0.000398 | 2.74E-66 | Pass | Yes | Yes | - |
| rs13230509 | 7 | 1286192 | C | G | 0.69 | -0.005525 | 0.000434 | 4.04E-37 | Pass | Yes | Yes | - |
| rs55759218 | 7 | 77453357 | A | G | 0.27 | -0.003904 | 0.0003869 | 6.09E-24 | Pass | Yes | Yes | - |
| rs700753 | 7 | 46753684 | C | G | 0.34 | 0.003295 | 0.0003613 | 7.50E-20 | Pass | Yes | Yes | - |
| rs2365286 | 7 | 156258179 | A | G | 0.74 | -0.003347 | 0.0003943 | 2.08E-17 | Pass | Yes | Yes | - |
| rs3757387 | 7 | 128576086 | T | C | 0.55 | 0.002914 | 0.0003555 | 2.48E-16 | Pass | Yes | Yes | - |
| rs6971211 | 7 | 155664686 | T | C | 0.41 | -0.002867 | 0.0003635 | 3.10E-15 | Pass | Yes | Yes | - |
| rs73116829 | 7 | 50739738 | A | G | 0.11 | -0.004256 | 0.0005771 | 1.64E-13 | Pass | Yes | Yes | - |
| rs62491533 | 7 | 129564134 | T | C | 0.83 | -0.002741 | 0.0004576 | 2.11E-09 | Pass | Yes | Yes | - |
| rs325442 | 7 | 127457228 | A | G | 0.4 | 0.002074 | 0.0003498 | 3.02E-09 | Pass | Yes | Yes | - |
| rs4871905 | 8 | 23735047 | C | G | 0.42 | -0.004303 | 0.0003462 | 1.82E-35 | Remove. Palindromic SNP with intermediate allele frequency | NA | NA | - |
| rs10086569 | 8 | 87247209 | T | C | 0.24 | 0.002774 | 0.0004029 | 5.73E-12 | Pass | Yes | Yes | - |
| rs2954017 | 8 | 126476873 | T | C | 0.46 | 0.002635 | 0.000397 | 3.18E-11 | Pass | Yes | No, significant associations with confounders identified in UK Biobank GWAS | Body mass index (UKB_ID:21001);Cholesterol-lowering medication (UKB_ID:6153_1);Cholesterol-lowering medication (UKB_ID:6177_1) |
| rs78936994 | 8 | 120894208 | T | G | 0.22 | 0.002425 | 0.0004234 | 1.02E-08 | Pass | Yes | Yes | - |
| rs7838146 | 8 | 22492143 | T | C | 0.36 | -0.002085 | 0.0003655 | 1.18E-08 | Pass | Yes | Yes | - |
| rs2039424 | 9 | 71432174 | A | G | 0.62 | 0.004828 | 0.0003613 | 9.75E-41 | Pass | Yes | Yes | - |
| rs4836732 | 9 | 119266695 | T | C | 0.53 | 0.002518 | 0.0003481 | 4.69E-13 | Pass | Yes | Yes | - |
| rs80282103 | 10 | 899071 | A | T | 0.92 | 0.008084 | 0.0006333 | 2.58E-37 | Pass | Yes | Yes | - |
| rs10994860 | 10 | 52645424 | T | C | 0.19 | 0.003891 | 0.000446 | 2.70E-18 | Pass | Yes | Yes | - |
| rs2068888 | 10 | 94839642 | A | G | 0.45 | -0.002622 | 0.0003496 | 6.31E-14 | Pass | Yes | Yes | - |
| rs7095954 | 10 | 82209232 | A | T | 0.47 | -0.001914 | 0.0003447 | 2.82E-08 | Remove. Not found in the LD reference. | NA | NA | - |
| rs10430743 | 10 | 126456997 | T | G | 0.43 | 0.002525 | 0.0003458 | 2.86E-13 | Pass | Yes | Yes | - |
| rs6481598 | 10 | 29781798 | C | G | 0.78 | 0.002313 | 0.0004204 | 3.77E-08 | Pass | Yes | Yes | - |
| rs3925584 | 11 | 30760335 | T | C | 0.55 | -0.005472 | 0.0003463 | 3.01E-56 | Pass | Yes | Yes | - |
| rs233438 | 11 | 2794392 | A | G | 0.81 | 0.004284 | 0.0004414 | 2.84E-22 | Pass | Yes | Yes | - |
| rs11227260 | 11 | 65461158 | T | G | 0.35 | -0.00322 | 0.0003608 | 4.47E-19 | Pass | Yes | Yes | - |
| rs11564722 | 11 | 2178330 | T | C | 0.24 | 0.00382 | 0.0004343 | 1.41E-18 | Pass | Yes | Yes | - |
| rs6484504 | 11 | 31424823 | T | C | 0.28 | -0.00323 | 0.0003899 | 1.17E-16 | Pass | Yes | Yes | - |
| rs10838702 | 11 | 47410888 | T | G | 0.38 | -0.002315 | 0.0003542 | 6.31E-11 | Pass | Yes | No, significant associations with confounders identified in UK Biobank GWAS | Body mass index (UKB_ID:21001) |
| rs3018667 | 11 | 68912221 | A | G | 0.32 | -0.002375 | 0.000369 | 1.23E-10 | Pass | Yes | Yes | - |
| rs12361687 | 11 | 9890052 | A | G | 0.36 | 0.002134 | 0.000365 | 5.03E-09 | Pass | Yes | Yes | - |
| rs10846157 | 12 | 15325031 | A | C | 0.81 | -0.003612 | 0.0004367 | 1.34E-16 | Pass | Yes | Yes | - |
| rs7974833 | 12 | 57791833 | T | C | 0.76 | -0.003224 | 0.0004104 | 3.97E-15 | Pass | Yes | Yes | - |
| rs7966357 | 12 | 51209838 | C | G | 0.66 | 0.002394 | 0.0003659 | 6.00E-11 | Pass | Yes | Yes | - |
| rs117113238 | 12 | 12209203 | A | G | 0.095 | 0.003939 | 0.0006099 | 1.06E-10 | Pass | Yes | Yes | - |
| rs17696736 | 12 | 112486818 | A | G | 0.57 | 0.002029 | 0.0003538 | 9.72E-09 | Pass | Yes | No, significant associations with confounders identified in UK Biobank GWAS | Ever smoked (UKB_ID:20160) |
| rs10774625 | 12 | 111910219 | A | G | 0.48 | -0.001964 | 0.0003485 | 1.76E-08 | Pass | Yes | No, significant associations with confounders identified in UK Biobank GWAS | Ever smoked (UKB_ID:20160) |
| rs11063193 | 12 | 4591100 | T | C | 0.88 | 0.002873 | 0.0005268 | 4.95E-08 | Pass | Yes | Yes | - |
| rs303937 | 13 | 72372524 | A | T | 0.41 | 0.002709 | 0.0003563 | 2.90E-14 | Pass | Yes | Yes | - |
| rs41284816 | 13 | 50655989 | T | G | 0.026 | -0.007874 | 0.0012304 | 1.56E-10 | Pass | Yes | Yes | - |
| rs7326821 | 13 | 96068204 | A | G | 0.83 | 0.002569 | 0.0004673 | 3.82E-08 | Pass | Yes | Yes | - |
| rs10851885 | 15 | 76304503 | A | G | 0.76 | 0.004972 | 0.0004077 | 3.28E-34 | Pass | Yes | Yes | - |
| rs2472297 | 15 | 75027880 | T | C | 0.26 | 0.003857 | 0.0004234 | 8.21E-20 | Pass | Yes | No, significant associations with confounders identified in UK Biobank GWAS | Body mass index (UKB_ID:21001) |
| rs6492982 | 15 | 41399951 | T | C | 0.55 | -0.00319 | 0.0003586 | 5.80E-19 | Pass | Yes | Yes | - |
| rs10851543 | 15 | 53962748 | A | G | 0.56 | 0.003048 | 0.0003463 | 1.34E-18 | Pass | Yes | Yes | - |
| rs4886699 | 15 | 75692303 | A | C | 0.75 | 0.003142 | 4.00E-04 | 4.00E-15 | Pass | Yes | Yes | - |
| rs12913015 | 15 | 39305443 | T | C | 0.44 | 0.002771 | 0.0003535 | 4.62E-15 | Pass | Yes | Yes | - |
| rs11071738 | 15 | 63580155 | T | C | 0.53 | -0.00249 | 0.0003452 | 5.49E-13 | Pass | Yes | Yes | - |
| rs506000 | 15 | 76817788 | T | C | 0.91 | -0.003825 | 0.0006123 | 4.17E-10 | Pass | Yes | Yes | - |
| rs1994887 | 15 | 57793765 | A | C | 0.28 | -0.002361 | 0.0003954 | 2.34E-09 | Pass | Yes | Yes | - |
| rs77924615 | 16 | 20392332 | A | G | 0.2 | 0.009576 | 0.0004519 | 1.21E-99 | Pass | Yes | Yes | - |
| rs154656 | 16 | 89708003 | A | T | 0.44 | -0.003079 | 0.0003497 | 1.32E-18 | Remove. Palindromic SNP with intermediate allele frequency | NA | NA | - |
| rs12920176 | 16 | 51761084 | A | C | 0.59 | -0.002618 | 0.0003573 | 2.35E-13 | Pass | Yes | Yes | - |
| rs7203398 | 16 | 53189672 | A | C | 0.73 | 0.002729 | 0.0003907 | 2.86E-12 | Pass | Yes | Yes | - |
| rs28581385 | 16 | 79942679 | A | T | 0.85 | -0.00328 | 0.0004866 | 1.58E-11 | Pass | Yes | Yes | - |
| rs1858800 | 16 | 73024276 | T | C | 0.35 | 0.002161 | 0.0003664 | 3.66E-09 | Pass | Yes | Yes | - |
| rs72817412 | 16 | 89141490 | T | C | 0.053 | 0.004865 | 0.0008599 | 1.54E-08 | Pass | Yes | Yes | - |
| rs56140069 | 16 | 69795323 | A | T | 0.82 | 0.002529 | 0.0004548 | 2.67E-08 | Pass | Yes | Yes | - |
| rs11657044 | 17 | 59450105 | T | C | 0.17 | -0.00755 | 0.0004621 | 5.34E-60 | Pass | Yes | Yes | - |
| rs9907229 | 17 | 58917399 | T | C | 0.85 | -0.004909 | 0.000487 | 6.83E-24 | Pass | Yes | Yes | - |
| rs2411192 | 17 | 34882998 | A | T | 0.59 | -0.002403 | 0.0003491 | 5.85E-12 | Pass | Yes | No, significant associations with confounders identified in UK Biobank GWAS | Body mass index (UKB_ID:21001) |
| rs9894634 | 17 | 1967501 | T | C | 0.6 | -0.002107 | 0.0003497 | 1.69E-09 | Pass | Yes | Yes | - |
| rs8096658 | 18 | 77156537 | C | G | 0.51 | 0.004558 | 0.0004043 | 1.77E-29 | Remove. Palindromic SNP with intermediate allele frequency | NA | NA | - |
| rs2337143 | 18 | 46482070 | A | G | 0.34 | -0.002148 | 0.0003686 | 5.59E-09 | Pass | Yes | Yes | - |
| rs8101667 | 19 | 33402419 | T | C | 0.33 | 0.005007 | 0.0003625 | 2.19E-43 | Pass | Yes | Yes | - |
| rs113445505 | 19 | 38157969 | T | C | 0.37 | 0.003775 | 0.0003549 | 1.96E-26 | Pass | Yes | Yes | - |
| rs111827672 | 19 | 37649866 | A | T | 0.32 | 0.003064 | 0.0003691 | 1.02E-16 | Pass | Yes | Yes | - |
| rs281380 | 19 | 49214470 | T | C | 0.63 | -0.002217 | 0.0003691 | 1.90E-09 | Pass | Yes | Yes | - |
| rs6127099 | 20 | 52731402 | A | T | 0.72 | -0.005128 | 0.0004055 | 1.17E-36 | Pass | Yes | Yes | - |
| rs2236521 | 20 | 60892116 | A | G | 0.55 | -0.002234 | 0.0003588 | 4.79E-10 | Pass | Yes | Yes | - |
| rs736820 | 20 | 43034016 | A | G | 0.37 | -0.002147 | 0.0003676 | 5.27E-09 | Pass | Yes | Yes | - |
| rs1570521 | 20 | 62911019 | T | G | 0.41 | 0.002029 | 0.0003524 | 8.54E-09 | Pass | Yes | Yes | - |
| rs62187541 | 20 | 1340244 | A | G | 0.93 | -0.003749 | 0.0006835 | 4.13E-08 | Pass | Yes | Yes | - |
| rs2823139 | 21 | 16576783 | A | G | 0.34 | -0.002714 | 0.0003648 | 1.01E-13 | Pass | Yes | Yes | - |
| rs2244237 | 21 | 37818141 | T | G | 0.22 | 0.00268 | 0.0004128 | 8.43E-11 | Pass | Yes | Yes | - |
| rs2834317 | 21 | 35356706 | A | G | 0.15 | -0.003091 | 0.00049 | 2.83E-10 | Pass | Yes | Yes | - |
| rs2267372 | 22 | 38598234 | A | G | 0.4 | 0.002444 | 0.0003608 | 1.25E-11 | Pass | Yes | Yes | - |

*SNP filters: filtering nonoverlap SNPs, ambiguous and palindromic SNPs with intermediate allele frequency.

**Table S6.** Summary of lead SNPs for the genetic instrument of kidney function based on serum creatinine eGFR refined by BUN

| rsID | Chr | Pos | Effect | Other | EAF  exposure | Beta  exposure | SE  exposure | P | SNP filters* | Selected for genetic instrument | Selected for conservative genetic instrument | Significant associations with confounders in UK Biobank |
| --- | --- | --- | --- | --- | --- | --- | --- | --- | --- | --- | --- | --- |
| rs267738 | 1 | 150940625 | T | G | 0.79 | -0.005007 | 0.0004211 | 1.33E-32 | Pass | Yes | Yes | - |
| rs6667182 | 1 | 15914545 | T | C | 0.32 | -0.004284 | 0.00043 | 2.22E-23 | Pass | Yes | Yes | - |
| rs1887252 | 1 | 82957871 | C | G | 0.64 | -0.002889 | 0.0003584 | 7.45E-16 | Pass | Yes | Yes | - |
| rs7543734 | 1 | 94050911 | C | G | 0.2 | 0.00312 | 0.000482 | 9.58E-11 | Pass | Yes | Yes | - |
| rs6546869 | 2 | 73895765 | A | G | 0.22 | 0.006087 | 0.0004159 | 1.66E-48 | Pass | Yes | Yes | - |
| rs807624 | 2 | 15782471 | T | G | 0.34 | 0.003376 | 0.0003633 | 1.53E-20 | Pass | Yes | Yes | - |
| rs187355703 | 2 | 176993583 | C | G | 0.97 | 0.010107 | 0.0011431 | 9.45E-19 | Pass | Yes | Yes | - |
| rs11694902 | 2 | 121988884 | A | G | 0.14 | 0.004132 | 0.000503 | 2.14E-16 | Pass | Yes | Yes | - |
| rs3791221 | 2 | 226933 | A | G | 0.65 | 0.002138 | 0.0003604 | 3.00E-09 | Pass | Yes | Yes | - |
| rs17462630 | 2 | 219286541 | C | G | 0.34 | 0.002406 | 0.0004097 | 4.32E-09 | Pass | Yes | Yes | - |
| rs112545201 | 3 | 185803532 | T | C | 0.13 | -0.004215 | 0.0005066 | 8.79E-17 | Pass | Yes | No, significant associations with confounders identified in UK Biobank GWAS | Body mass index (UKB_ID:21001) |
| rs9868185 | 3 | 121657593 | A | G | 0.54 | 0.002653 | 0.0003451 | 1.50E-14 | Pass | Yes | Yes | - |
| rs3905668 | 3 | 135931586 | A | G | 0.72 | -0.00255 | 0.000383 | 2.81E-11 | Pass | Yes | No, significant associations with confounders identified in UK Biobank GWAS | Body mass index (UKB_ID:21001) |
| rs9828976 | 3 | 136536835 | C | G | 0.75 | -0.002385 | 0.0003978 | 2.04E-09 | Pass | Yes | No, significant associations with confounders identified in UK Biobank GWAS | Body mass index (UKB_ID:21001) |
| rs28817415 | 4 | 77401452 | T | C | 0.44 | -0.007437 | 0.0003438 | 9.68E-104 | Remove. Not found in the outcome dataset. | NA | NA | - |
| rs1458038 | 4 | 81164723 | T | C | 0.3 | 0.003197 | 0.0003795 | 3.60E-17 | Pass | Yes | No, significant associations with confounders identified in UK Biobank GWAS | Cholesterol-lowering medication (UKB_ID:6177_1) |
| rs3812036 | 5 | 176813404 | T | C | 0.26 | -0.006869 | 0.0004059 | 3.19E-64 | Pass | Yes | Yes | - |
| rs79760705 | 5 | 53298716 | T | G | 0.11 | 0.005609 | 0.0005512 | 2.55E-24 | Pass | Yes | Yes | - |
| rs13157326 | 5 | 34504277 | A | G | 0.48 | -0.002706 | 0.0003877 | 2.95E-12 | Pass | Yes | Yes | - |
| rs13200335 | 6 | 41690823 | A | C | 0.42 | 0.002361 | 0.0003498 | 1.50E-11 | Pass | Yes | Yes | - |
| rs10224002 | 7 | 151415041 | A | G | 0.72 | 0.006845 | 0.000398 | 2.74E-66 | Pass | Yes | Yes | - |
| rs13230509 | 7 | 1286192 | C | G | 0.69 | -0.005525 | 0.000434 | 4.04E-37 | Pass | Yes | Yes | - |
| rs700753 | 7 | 46753684 | C | G | 0.34 | 0.003295 | 0.0003613 | 7.50E-20 | Pass | Yes | Yes | - |
| rs4410790 | 7 | 17284577 | T | C | 0.37 | -0.002286 | 0.000359 | 1.92E-10 | Pass | Yes | Yes | - |
| rs6948759 | 7 | 33095688 | T | C | 0.21 | -0.002577 | 0.000422 | 1.01E-09 | Pass | Yes | Yes | - |
| rs2068888 | 10 | 94839642 | A | G | 0.45 | -0.002622 | 0.0003496 | 6.31E-14 | Pass | Yes | Yes | - |
| rs3925584 | 11 | 30760335 | T | C | 0.55 | -0.005472 | 0.0003463 | 3.01E-56 | Pass | Yes | Yes | - |
| rs6484504 | 11 | 31424823 | T | C | 0.28 | -0.00323 | 0.0003899 | 1.17E-16 | Pass | Yes | Yes | - |
| rs10838702 | 11 | 47410888 | T | G | 0.38 | -0.002315 | 0.0003542 | 6.31E-11 | Pass | Yes | No, significant associations with confounders identified in UK Biobank GWAS | Body mass index (UKB_ID:21001) |
| rs10846157 | 12 | 15325031 | A | C | 0.81 | -0.003612 | 0.0004367 | 1.34E-16 | Pass | Yes | Yes | - |
| rs7974833 | 12 | 57791833 | T | C | 0.76 | -0.003224 | 0.0004104 | 3.97E-15 | Pass | Yes | Yes | - |
| rs117113238 | 12 | 12209203 | A | G | 0.095 | 0.003939 | 0.0006099 | 1.06E-10 | Pass | Yes | Yes | - |
| rs303937 | 13 | 72372524 | A | T | 0.41 | 0.002709 | 0.0003563 | 2.90E-14 | Pass | Yes | Yes | - |
| rs10851885 | 15 | 76304503 | A | G | 0.76 | 0.004972 | 0.0004077 | 3.28E-34 | Pass | Yes | Yes | - |
| rs2472297 | 15 | 75027880 | T | C | 0.26 | 0.003857 | 0.0004234 | 8.21E-20 | Pass | Yes | No, significant associations with confounders identified in UK Biobank GWAS | Body mass index (UKB_ID:21001) |
| rs10851543 | 15 | 53962748 | A | G | 0.56 | 0.003048 | 0.0003463 | 1.34E-18 | Pass | Yes | Yes | - |
| rs506000 | 15 | 76817788 | T | C | 0.91 | -0.003825 | 0.0006123 | 4.17E-10 | Pass | Yes | Yes | - |
| rs77924615 | 16 | 20392332 | A | G | 0.2 | 0.009576 | 0.0004519 | 1.21E-99 | Pass | Yes | Yes | - |
| rs12920176 | 16 | 51761084 | A | C | 0.59 | -0.002618 | 0.0003573 | 2.35E-13 | Pass | Yes | Yes | - |
| rs7203398 | 16 | 53189672 | A | C | 0.73 | 0.002729 | 0.0003907 | 2.86E-12 | Pass | Yes | Yes | - |
| rs9907229 | 17 | 58917399 | T | C | 0.85 | -0.004909 | 0.000487 | 6.83E-24 | Pass | Yes | Yes | - |
| rs8096658 | 18 | 77156537 | C | G | 0.51 | 0.004558 | 0.0004043 | 1.77E-29 | Remove. Palindromic SNP with intermediate allele frequency | NA | NA | - |
| rs113445505 | 19 | 38157969 | T | C | 0.37 | 0.003775 | 0.0003549 | 1.96E-26 | Pass | Yes | Yes | - |
| rs111827672 | 19 | 37649866 | A | T | 0.32 | 0.003064 | 0.0003691 | 1.02E-16 | Pass | Yes | Yes | - |
| rs6127099 | 20 | 52731402 | A | T | 0.72 | -0.005128 | 0.0004055 | 1.17E-36 | Pass | Yes | Yes | - |
| rs2823139 | 21 | 16576783 | A | G | 0.34 | -0.002714 | 0.0003648 | 1.01E-13 | Pass | Yes | Yes | - |
| rs2244237 | 21 | 37818141 | T | G | 0.22 | 0.00268 | 0.0004128 | 8.43E-11 | Pass | Yes | Yes | - |

*SNP filters: filtering nonoverlap SNPs, ambiguous and palindromic SNPs with intermediate allele frequency.

**Table S7.** Findings of summary-based MR from genetically predicted EAA to eGFR and CKD based on the primary method and pleiotropy or outlier-robust methods

| Genetically predicted exposure | Outcome | Genetic instruments | SNP R^2^ for exposure* | SNP R^2^ for outcome* | Steiger test for directionality | Egger intercept P | Summary-level analysis method | F-statistic (IVW) or I^2^ (Egger) | beta (per 1-year EAA) | se (per 1-year EAA) | P | Effect per 1-year EAA (95% CI) | Effect per 5-year EAA (95% CI) |
| --- | --- | --- | --- | --- | --- | --- | --- | --- | --- | --- | --- | --- | --- |
| IEAA | Log(eGFR) | 36 SNPs | 0.0649 | 0.0001 | Correct direction | 0.02 | Inverse variance weighted | 54.73 | -0.0001 | 0.0004 | 0.74 | -0.0001 (-0.001,0.001) | -0.001 (-0.004,0.003) |
|  |  |  |  |  |  |  | MR Egger | 0.98 | -0.002 | 0.0008 | 0.02 | -0.002 (-0.003,-0.0003) | -0.01 (-0.02,-0.002) |
|  |  |  |  |  |  |  | Weighted median |  | -0.0001 | 0.0004 | 0.88 | -0.0001 (-0.001,0.001) | -0.0003 (-0.004,0.004) |
|  |  |  |  |  |  |  | MR-PRESSO |  | -0.0003 | 0.0003 | 0.36 | -0.0003 (-0.001,0.0003) | -0.001 (-0.005,0.002) |
| GrimAA | Log(eGFR) | 4 SNPs | 0.0047 | 0.0001 | Correct direction | 0.51 | Inverse variance weighted | 37.2 | -0.004 | 0.0026 | 0.09 | -0.004 (-0.01,0.001) | -0.02 (-0.05,0.004) |
|  |  |  |  |  |  |  | MR Egger | 0.98 | 0.03 | 0.0463 | 0.56 | 0.03 (-0.06,0.12) | 0.16 (-0.29,0.62) |
|  |  |  |  |  |  |  | Weighted median |  | -0.004 | 0.0014 | 0.01 | -0.004 (-0.01,-0.001) | -0.02 (-0.03,-0.004) |
|  |  |  |  |  |  |  | MR-PRESSO |  | -0.0037 | 0.0004 | 0.07 | -0.004 (-0.005,-0.003) | -0.02 (-0.02,-0.01) |
| PhenoAA | Log(eGFR) | 13 SNPs | 0.0220 | 0.0001 | Correct direction | 0.92 | Inverse variance weighted | 52.46 | -0.0001 | 0.0007 | 0.86 | -0.0001 (-0.004,0.003) | -0.001 (-0.01,0.01) |
|  |  |  |  |  |  |  | MR Egger | 0.98 | -0.0003 | 0.0021 | 0.88 | -0.0003 (-0.004,0.004) | -0.002 (-0.02,0.02) |
|  |  |  |  |  |  |  | Weighted median |  | 0.00002 | 0.0004 | 0.96 | 0.00002 (-0.001,0.001) | 0.0001 (-0.004,0.004) |
|  |  |  |  |  |  |  | MR-PRESSO |  | 0.00004 | 0.0002 | 0.88 | 0.00004 (-0.0004,0.0005) | 0.0002 (-0.002,0.002) |
| HannumAA | Log(eGFR) | 22 SNPs | 0.0371 | 0.0001 | Correct direction | 0.72 | Inverse variance weighted | 47.74 | 0.0004 | 0.0007 | 0.52 | 0.0004 (-0.001,0.002) | 0.002 (-0.005,0.01) |
|  |  |  |  |  |  |  | MR Egger | 0.98 | -0.0001 | 0.0017 | 0.94 | -0.0001 (-0.004,0.003) | -0.001 (-0.02,0.02) |
|  |  |  |  |  |  |  | Weighted median |  | 0.001 | 0.0005 | 0.15 | 0.001 (-0.0003,0.002) | 0.004 (-0.001,0.01) |
|  |  |  |  |  |  |  | MR-PRESSO |  | 0.0004 | 0.0004 | 0.25 | 0.0004 (-0.0003,0.001) | 0.002 (-0.001,0.01) |
| IEAA | CKD | 36 SNPs | 0.0649 | 0.0002 | Correct direction | 0.05 | Inverse variance weighted | 54.73 | 0.004 | 0.0099 | 0.71 | 1 (0.98,1.02) | 1.02 (0.92,1.12) |
|  |  |  |  |  |  |  | MR Egger | 0.98 | 0.043 | 0.0218 | 0.06 | 1.04 (1,1.09) | 1.24 (1,1.54) |
|  |  |  |  |  |  |  | Weighted median |  | -0.002 | 0.0103 | 0.88 | 1 (0.98,1.02) | 0.99 (0.9,1.1) |
|  |  |  |  |  |  |  | MR-PRESSO |  | 0.010 | 0.0082 | 0.24 | 1.01 (0.99,1.03) | 1.05 (0.97,1.14) |
| GrimAA | CKD | 4 SNPs | 0.0047 | 0.0000 | Correct direction | 0.41 | Inverse variance weighted | 37.2 | 0.03 | 0.0352 | 0.35 | 1.0337 (0.96,1.11) | 1.18 (0.84,1.67) |
|  |  |  |  |  |  |  | MR Egger | 0.98 | 0.63 | 0.5765 | 0.39 | 1.88 (0.61,5.83) | 23.67 (0.08,6730.62) |
|  |  |  |  |  |  |  | Weighted median |  | 0.05 | 0.0313 | 0.13 | 1.05 (0.99,1.11) | 1.26 (0.93,1.72) |
|  |  |  |  |  |  |  | MR-PRESSO** |  | NA | NA | NA | NA | NA |
| PhenoAA | CKD | 13 SNPs | 0.0220 | 0.0001 | Correct direction | 0.36 | Inverse variance weighted | 52.46 | -0.01 | 0.0154 | 0.46 | 0.99 (0.96,1.02) | 0.94 (0.81,1.1) |
|  |  |  |  |  |  |  | MR Egger | 0.98 | 0.03 | 0.0453 | 0.53 | 1.03 (0.94,1.13) | 1.16 (0.74,1.81) |
|  |  |  |  |  |  |  | Weighted median |  | -0.01 | 0.0129 | 0.54 | 0.99 (0.97,1.02) | 0.96 (0.85,1.09) |
|  |  |  |  |  |  |  | MR-PRESSO |  | -0.01 | 0.0073 | 0.13 | 0.99 (0.97,1) | 0.94 (0.88,1.01) |
| HannumAA | CKD | 22 SNPs | 0.0371 | 0.0001 | Correct direction | 0.10 | Inverse variance weighted | 47.74 | -0.02 | 0.0134 | 0.25 | 0.98 (0.96,1.01) | 0.93 (0.81,1.06) |
|  |  |  |  |  |  |  | MR Egger | 0.98 | 0.03 | 0.0321 | 0.29 | 1.04 (0.97,1.1) | 1.19 (0.87,1.63) |
|  |  |  |  |  |  |  | Weighted median |  | 0.003 | 0.0139 | 0.82 | 1 (0.98,1.03) | 1.02 (0.89,1.16) |
|  |  |  |  |  |  |  | MR-PRESSO |  | -0.01 | 0.0091 | 0.47 | 0.99 (0.98,1.01) | 0.97 (0.88,1.06) |

* R^2^ values are approximate for Steiger test.

**Indicates the global test of MR-PRESSO detected no significant outlier instruments and no correction was performed.

**Table S8.** Findings of summary-based MR sensitivity analysis using conservative genetic instruments from genetically predicted EAA to eGFR and CKD based on the primary method and pleiotropy or outline-robust methods

| Genetically predicted exposure | Outcome | Genetic instruments | SNP R^2^ for exposure* | SNP R^2^ for outcome* | Steiger test for directionality | Egger intercept P | Summary-level analysis method | F-statistic (IVW) or I^2^ (Egger) | beta (per 1-year EAA) | se (per 1-year EAA) | P | Effect per 1-year EAA (95% CI) | Effect per 5-year EAA (95% CI) |
| --- | --- | --- | --- | --- | --- | --- | --- | --- | --- | --- | --- | --- | --- |
| IEAA | Log(eGFR) | 22 SNPs | 0.0458 | 0.0001 | Correct direction | 0.07 | Inverse variance weighted | 60.24 | -0.0004 | 0.0004 | 0.26 | -0.0004 (-0.001,0.0003) | -0.002 (-0.006,0.002) |
|  |  |  |  |  |  |  | MR Egger | 0.98 | -0.0017 | 0.0007 | 0.03 | -0.002 (-0.003,-0.0002) | -0.01 (-0.02,-0.001) |
|  |  |  |  |  |  |  | Weighted median |  | -0.0003 | 0.0005 | 0.45 | -0.0003 (-0.001,0.001) | -0.002 (-0.01,0.003) |
|  |  |  |  |  |  |  | MR-PRESSO** |  | NA | NA | NA | NA | NA |
| GrimAA | Log(eGFR) | 3 SNPs | 0.0037 | 0.0001 | Correct direction | 0.18 | Inverse variance weighted | 39.17 | -0.0060 | 0.0026 | 0.02 | -0.01 (-0.01,-0.001) | -0.03 (-0.06,-0.005) |
|  |  |  |  |  |  |  | MR Egger | 0.98 | 0.0497 | 0.0166 | 0.20 | 0.05 (0.02,0.08) | 0.25 (0.09,0.41) |
|  |  |  |  |  |  |  | Weighted median |  | -0.0039 | 0.0015 | 0.01 | -0.004 (-0.01,-0.001) | -0.02 (-0.03,-0.005) |
|  |  |  |  |  |  |  | MR-PRESSO** |  | NA | NA | NA | NA | NA |
| PhenoAA | Log(eGFR) | 9 SNPs | 0.0170 | 0.0001 | Correct direction | 0.39 | Inverse variance weighted | 58.34 | -0.0006 | 0.0008 | 0.45 | -0.001 (-0.002,0.001) | -0.003 (-0.01,0.005) |
|  |  |  |  |  |  |  | MR Egger | 0.98 | 0.0015 | 0.0024 | 0.55 | 0.001 (-0.003,0.006) | 0.01 (-0.02,0.03) |
|  |  |  |  |  |  |  | Weighted median |  | 0.0000 | 0.0005 | 0.95 | -0.00003 (-0.001,0.001) | -0.0002 (-0.005,0.005) |
|  |  |  |  |  |  |  | MR-PRESSO** |  | NA | NA | NA | NA | NA |
| HannumAA | Log(eGFR) | 19 SNPs | 0.0336 | 0.00004 | Correct direction | 0.06 | Inverse variance weighted | 49.54 | 0.0005 | 0.0004 | 0.23 | 0.0005 (-0.0003,0.001) | 0.002 (-0.002,0.01) |
|  |  |  |  |  |  |  | MR Egger | 0.98 | -0.0013 | 0.0009 | 0.20 | -0.001 (-0.003,0.001) | -0.01 (-0.02,0.003) |
|  |  |  |  |  |  |  | Weighted median |  | 0.0007 | 0.0005 | 0.16 | 0.001 (-0.0003,0.002) | 0.004 (-0.001,0.01) |
|  |  |  |  |  |  |  | MR-PRESSO** |  | NA | NA | NA | NA | NA |
| IEAA | CKD | 22 SNPs | 0.0458 | 0.0001 | Correct direction | 0.04 | Inverse variance weighted | 60.24 | 0.0126 | 0.0101 | 0.21 | 1.01 (0.99,1.03) | 1.06 (0.96,1.18) |
|  |  |  |  |  |  |  | MR Egger | 0.98 | 0.0513 | 0.0203 | 0.02 | 1.05 (1.01,1.1) | 1.29 (1.06,1.58) |
|  |  |  |  |  |  |  | Weighted median |  | -0.0009 | 0.0124 | 0.94 | 1 (0.98,1.02) | 1 (0.88,1.12) |
|  |  |  |  |  |  |  | MR-PRESSO** |  | NA | NA | NA | NA | NA |
| GrimAA | CKD | 3 SNPs | 0.0037 | 0.00001 | Correct direction | 0.55 | Inverse variance weighted | 39.17 | 0.0611 | 0.0287 | 0.03 | 1.063 (1,1.12) | 1.36 (1.02,1.8) |
|  |  |  |  |  |  |  | MR Egger | 0.98 | 0.4277 | 0.4399 | 0.51 | 1.53 (0.65,3.63) | 8.49 (0.11,632.18) |
|  |  |  |  |  |  |  | Weighted median |  | 0.0631 | 0.0373 | 0.09 | 1.07 (0.99,1.15) | 1.37 (0.95,1.98) |
|  |  |  |  |  |  |  | MR-PRESSO** |  | NA | NA | NA | NA | NA |
| PhenoAA | CKD | 9 SNPs | 0.0170 | 0.0001 | Correct direction | 0.91 | Inverse variance weighted | 58.34 | 0.0031 | 0.0153 | 0.84 | 1 (0.97,1.03) | 1.02 (0.87,1.18) |
|  |  |  |  |  |  |  | MR Egger | 0.98 | -0.0020 | 0.0489 | 0.97 | 1 (0.91,1.1) | 0.99 (0.61,1.6) |
|  |  |  |  |  |  |  | Weighted median |  | -0.0013 | 0.0140 | 0.93 | 1 (0.97,1.03) | 0.99 (0.87,1.14) |
|  |  |  |  |  |  |  | MR-PRESSO** |  | NA | NA | NA | NA | NA |
| HannumAA | CKD | 19 SNPs | 0.0336 | 0.00004 | Correct direction | 0.15 | Inverse variance weighted | 49.54 | -0.0098 | 0.0105 | 0.35 | 0.99 (0.97,1.01) | 0.95 (0.86,1.06) |
|  |  |  |  |  |  |  | MR Egger | 0.98 | 0.0269 | 0.0263 | 0.32 | 1.03 (0.98,1.08) | 1.14 (0.88,1.48) |
|  |  |  |  |  |  |  | Weighted median |  | 0.0004 | 0.0140 | 0.98 | 1 (0.97,1.03) | 1 (0.87,1.15) |
|  |  |  |  |  |  |  | MR-PRESSO** |  | NA | NA | NA | NA | NA |

* R^2^ values are approximate for Steiger test.

**Indicates the global test of MR-PRESSO detected no significant outlier instruments and no correction was performed.

**Table S9.** Findings of summary-based MR from genetically predicted eGFR to EAA based on the primary method and pleiotropy or outlier-robust methods

| Genetically predicted exposure | Outcome (1-year) | Genetic instruments | SNP R^2^ for exposure* | SNP R^2^ for outcome* | Steiger test for directionality | Egger intercept P | Summary-level analysis method | F-statistic (IVW) or I^2^ (Egger) | beta | se | P | Effect (95% CI) |
| --- | --- | --- | --- | --- | --- | --- | --- | --- | --- | --- | --- | --- |
| eGFRcr + eGFRcys | IEAA | 133 SNPs | 0.0186 | 0.0057 | Correct direction | 0.91 | MR Egger | 0.99 | -1.82 | 2.60 | 0.49 | -1.82 (-6.92,3.28) |
|  |  |  |  |  |  |  | Weighted median |  | -0.83 | 1.45 | 0.57 | -0.83 (-3.67,2.01) |
|  |  |  |  |  |  |  | Inverse variance weighted | 74.86 | -1.55 | 0.99 | 0.12 | -1.55 (-3.5,0.4) |
|  |  |  |  |  |  |  | MR-PRESSO |  | -1.77 | 0.94 | 0.06 | -1.77 (-3.61,0.07) |
| eGFRcr + eGFRcys | HannumAA | 133 SNPs | 0.0186 | 0.0074 | Correct direction | 0.23 | MR Egger | 0.99 | -0.07 | 2.78 | 0.98 | -0.07 (-5.52,5.37) |
|  |  |  |  |  |  |  | Weighted median |  | -2.24 | 1.40 | 0.11 | -2.24 (-4.98,0.5) |
|  |  |  |  |  |  |  | Inverse variance weighted | 74.86 | -3.14 | 1.07 | 0.00 | -3.14 (-5.23,-1.05) |
|  |  |  |  |  |  |  | MR-PRESSO |  | -2.81 | 1.01 | 0.01 | -2.81 (-4.79,-0.83) |
| eGFRcr + eGFRcys | GrimAA | 133 SNPs | 0.0186 | 0.0059 | Correct direction | 0.17 | MR Egger | 0.99 | 1.34 | 2.59 | 0.61 | 1.34 (-3.74,6.42) |
|  |  |  |  |  |  |  | Weighted median |  | -1.27 | 1.43 | 0.37 | -1.27 (-4.08,1.53) |
|  |  |  |  |  |  |  | Inverse variance weighted | 74.86 | -1.99 | 0.99 | 0.05 | -1.99 (-3.94,-0.04) |
|  |  |  |  |  |  |  | MR-PRESSO** |  | NA | NA | NA | NA |
| eGFRcr + eGFRcys | PhenoAA | 133 SNPs | 0.0186 | 0.0054 | Correct direction | 0.40 | MR Egger | 0.99 | -0.43 | 3.12 | 0.89 | -0.43 (-6.55,5.69) |
|  |  |  |  |  |  |  | Weighted median |  | -2.44 | 1.80 | 0.18 | -2.44 (-5.96,1.09) |
|  |  |  |  |  |  |  | Inverse variance weighted | 74.86 | -2.88 | 1.19 | 0.02 | -2.88 (-5.21,-0.54) |
|  |  |  |  |  |  |  | MR-PRESSO** |  | NA | NA | NA | NA |
| eGFRcr + BUN | IEAA | 45 SNPs | 0.0083 | 0.0019 | Correct direction | 0.98 | MR Egger | 0.99 | -2.18 | 3.89 | 0.58 | -2.18 (-9.8,5.44) |
|  |  |  |  |  |  |  | Weighted median |  | -1.16 | 2.02 | 0.57 | -1.16 (-5.11,2.79) |
|  |  |  |  |  |  |  | Inverse variance weighted | 97.71 | -2.08 | 1.47 | 0.16 | -2.08 (-4.95,0.79) |
|  |  |  |  |  |  |  | MR-PRESSO** |  | NA | NA | NA | NA |
| eGFRcr + BUN | HannumAA | 45 SNPs | 0.0083 | 0.0022 | Correct direction | 0.35 | MR Egger | 0.99 | 0.16 | 3.89 | 0.97 | 0.16 (-7.47,7.79) |
|  |  |  |  |  |  |  | Weighted median |  | -1.93 | 1.98 | 0.33 | -1.93 (-5.82,1.96) |
|  |  |  |  |  |  |  | Inverse variance weighted | 97.71 | -3.24 | 1.48 | 0.03 | -3.24 (-6.15,-0.34) |
|  |  |  |  |  |  |  | MR-PRESSO** |  | NA | NA | NA | NA |
| eGFRcr + BUN | GrimAA | 45 SNPs | 0.0083 | 0.0017 | Correct direction | 0.53 | MR Egger | 0.99 | 0.75 | 3.51 | 0.83 | 0.75 (-6.12,7.62) |
|  |  |  |  |  |  |  | Weighted median |  | -0.87 | 1.92 | 0.65 | -0.87 (-4.64,2.9) |
|  |  |  |  |  |  |  | Inverse variance weighted | 97.71 | -1.31 | 1.32 | 0.32 | -1.31 (-3.9,1.29) |
|  |  |  |  |  |  |  | MR-PRESSO** |  | NA | NA | NA | NA |
| eGFRcr + BUN | PhenoAA | 45 SNPs | 0.0083 | 0.0017 | Correct direction | 0.90 | MR Egger | 0.99 | -1.41 | 4.63 | 0.76 | -1.41 (-10.5,7.67) |
|  |  |  |  |  |  |  | Weighted median |  | -2.55 | 2.52 | 0.31 | -2.55 (-7.5,2.4) |
|  |  |  |  |  |  |  | Inverse variance weighted | 97.71 | -0.88 | 1.74 | 0.62 | -0.88 (-4.29,2.54) |
|  |  |  |  |  |  |  | MR-PRESSO** |  | NA | NA | NA | NA |

* R^2^ values are approximate for Steiger test.

**Indicates the global test of MR-PRESSO detected no significant outlier instruments and no correction was performed.

**Table S10.** Findings of summary-based MR sensitivity analysis using conservative genetic instruments from genetically predicted eGFR to EAA based on the primary method and pleiotropy or outline-robust methods

| Genetically predicted exposure | Outcome (1-year) | Genetic instruments | SNP R^2^ for exposure* | SNP R^2^ for outcome* | Steiger test for directionality | Egger intercept P | Summary-level analysis method | F-statistic (IVW) or I^2^ (Egger) | beta | se | P | Effect (95% CI) |
| --- | --- | --- | --- | --- | --- | --- | --- | --- | --- | --- | --- | --- |
| eGFRcr + eGFRcys | IEAA | 119 SNPs | 0.0171 | 0.0054 | Correct direction | 0.78 | MR Egger | 0.99 | -2.28 | 2.75 | 0.41 | -2.28 (-7.66,3.1) |
|  |  |  |  |  |  |  | Weighted median |  | -1.06 | 1.48 | 0.47 | -1.06 (-3.97,1.85) |
|  |  |  |  |  |  |  | Inverse variance weighted | 76.72 | -1.57 | 1.07 | 0.14 | -1.57 (-3.66,0.52) |
|  |  |  |  |  |  |  | MR-PRESSO |  | -1.82 | 1.00 | 0.07 | -1.82 (-3.78,0.15) |
| eGFRcr + eGFRcys | HannumAA | 119 SNPs | 0.0171 | 0.0067 | Correct direction | 0.24 | MR Egger | 0.99 | 0.37 | 2.89 | 0.90 | 0.37 (-5.29,6.04) |
|  |  |  |  |  |  |  | Weighted median |  | -2.14 | 1.52 | 0.16 | -2.14 (-5.12,0.84) |
|  |  |  |  |  |  |  | Inverse variance weighted | 76.72 | -2.78 | 1.13 | 0.01 | -2.78 (-4.99,-0.57) |
|  |  |  |  |  |  |  | MR-PRESSO |  | -2.42 | 1.06 | 0.03 | -2.42 (-4.5,-0.33) |
| eGFRcr + eGFRcys | GrimAA | 119 SNPs | 0.0171 | 0.0055 | Correct direction | 0.40 | MR Egger | 0.99 | 0.35 | 2.72 | 0.90 | 0.35 (-4.99,5.69) |
|  |  |  |  |  |  |  | Weighted median |  | -1.35 | 1.52 | 0.38 | -1.35 (-4.33,1.63) |
|  |  |  |  |  |  |  | Inverse variance weighted | 76.72 | -1.79 | 1.06 | 0.09 | -1.79 (-3.87,0.28) |
|  |  |  |  |  |  |  | MR-PRESSO** |  | NA | NA | NA | NA |
| eGFRcr + eGFRcys | PhenoAA | 119 SNPs | 0.0171 | 0.0049 | Correct direction | 0.73 | MR Egger | 0.99 | -1.81 | 3.23 | 0.58 | -1.81 (-8.14,4.52) |
|  |  |  |  |  |  |  | Weighted median |  | -2.76 | 1.95 | 0.16 | -2.76 (-6.59,1.07) |
|  |  |  |  |  |  |  | Inverse variance weighted | 76.72 | -2.83 | 1.25 | 0.02 | -2.83 (-5.28,-0.37) |
|  |  |  |  |  |  |  | MR-PRESSO** |  | NA | NA | NA | NA |
| eGFRcr + BUN | IEAA | 39 SNPs | 0.0077 | 0.0017 | Correct direction | 0.83 | MR Egger | 0.99 | -2.82 | 4.10 | 0.50 | -2.82 (-10.85,5.22) |
|  |  |  |  |  |  |  | Weighted median |  | -0.83 | 2.09 | 0.69 | -0.83 (-4.93,3.28) |
|  |  |  |  |  |  |  | Inverse variance weighted | 103.58 | -1.99 | 1.56 | 0.20 | -1.99 (-5.06,1.07) |
|  |  |  |  |  |  |  | MR-PRESSO** |  | NA | NA | NA | NA |
| eGFRcr + BUN | HannumAA | 39 SNPs | 0.0077 | 0.0021 | Correct direction | 0.37 | MR Egger | 0.99 | 0.22 | 4.19 | 0.96 | 0.22 (-7.99,8.42) |
|  |  |  |  |  |  |  | Weighted median |  | -2.16 | 2.23 | 0.33 | -2.16 (-6.53,2.21) |
|  |  |  |  |  |  |  | Inverse variance weighted | 103.58 | -3.27 | 1.61 | 0.04 | -3.27 (-6.43,-0.11) |
|  |  |  |  |  |  |  | MR-PRESSO** |  | NA | NA | NA | NA |
| eGFRcr + BUN | GrimAA | 39 SNPs | 0.0077 | 0.0015 | Correct direction | 0.52 | MR Egger | 0.99 | 0.65 | 3.67 | 0.86 | 0.65 (-6.54,7.84) |
|  |  |  |  |  |  |  | Weighted median |  | -1.00 | 2.02 | 0.62 | -1 (-4.96,2.97) |
|  |  |  |  |  |  |  | Inverse variance weighted | 103.58 | -1.53 | 1.40 | 0.28 | -1.53 (-4.28,1.22) |
|  |  |  |  |  |  |  | MR-PRESSO** |  | NA | NA | NA | NA |
| eGFRcr + BUN | PhenoAA | 39 SNPs | 0.0077 | 0.0016 | Correct direction | 0.81 | MR Egger | 0.99 | -1.67 | 4.89 | 0.74 | -1.67 (-11.26,7.93) |
|  |  |  |  |  |  |  | Weighted median |  | -2.65 | 2.64 | 0.32 | -2.65 (-7.83,2.52) |
|  |  |  |  |  |  |  | Inverse variance weighted | 103.58 | -0.59 | 1.86 | 0.75 | -0.59 (-4.24,3.06) |
|  |  |  |  |  |  |  | MR-PRESSO** |  | NA | NA | NA | NA |

* R^2^ values are approximate for Steiger test.

**Indicates the global test of MR-PRESSO detected no significant outlier instruments and no correction was performed.

**Table S11.** Multivariable MR and mediation analysis of lymphocyte count on the association of kidney function with HannumAA

*Multivariable MR to investigate the effect of kidney function (eGFR) and lymphocyte count on HannumAA*

| Multivariable MR | Exposures | Outcome | Instrumental SNPs (N) | Effect (95% CI) | P |
| --- | --- | --- | --- | --- | --- |
| Exposure 1 | eGFR | HannumAA | 127 | -1.95 (-3.86,-0.03) | 4.70E-02 |
| Exposure 2 | lymphocyte |  | 479 | -0.62 (-0.77,-0.46) | 6.60E-15 |

*Mediation analysis of lymphocyte count on the association of kidney function (eGFR) with HannumAA*

|  | Total effect  (95% CI) | Direct effect  (95% CI) | Indirect effect  (95% CI) | Proportion Mediated |
| --- | --- | --- | --- | --- |
| Indirect effect | -3.14 (-5.23,-1.05) | -1.95 (-3.86,-0.03) | -1.19 (-4.03,1.65) | 38% |

CI, confidence interval; eGFR, estimated glomerular filtration rate; MR, mendelian randomization; SNP, single nucleotide polymorphism.

**Table S12**. Power analysis of the two-sample MR using the Brion et al. method

| Outcome | Sample size | Proportion of variance explained for the association between the genetic instrument and exposure (R^2^) | True underlying causal association between the exposure and outcome variables | Power | Strength of the instrument |
| --- | --- | --- | --- | --- | --- |
| eGFR | 567460 | 0.0039 (GrimAA) | 0.03 | 0.29 | 2222.76 |
|  | 567460 | 0.0039 (GrimAA) | 0.04 | 0.47 | 2222.76 |
|  | 567460 | 0.0039 (GrimAA) | 0.05 | 0.65 | 2222.76 |
|  | **567460** | **0.0039 (GrimAA)** | **0.06** | **0.81** | **2222.76** |
|  | 567460 | 0.0113 (HannumAA) | 0.03 | 0.67 | 6486.59 |
|  | **567460** | **0.0113 (HannumAA)** | **0.04** | **0.89** | **6486.59** |
|  | **567460** | **0.0113 (HannumAA)** | **0.05** | **0.98** | **6486.59** |
|  | **567460** | **0.0113 (HannumAA)** | **0.06** | **1** | **6486.59** |
|  | 567460 | 0.0138 (PhenoAA) | 0.03 | 0.76 | 7941.53 |
|  | **567460** | **0.0138 (PhenoAA)** | **0.04** | **0.94** | **7941.53** |
|  | **567460** | **0.0138 (PhenoAA)** | **0.05** | **0.99** | **7941.53** |
|  | **567460** | **0.0138 (PhenoAA)** | **0.06** | **1** | **7941.53** |
|  | **567460** | **0.0272 (IEAA)** | **0.03** | **0.96** | **15867.5** |
|  | **567460** | **0.0272 (IEAA)** | **0.04** | **1** | **15867.5** |
|  | **567460** | **0.0272 (IEAA)** | **0.05** | **1** | **15867.5** |
|  | **567460** | **0.0272 (IEAA)** | **0.06** | **1** | **15867.5** |
| CKD | 480698 (8.61% cases) | 0.0039 (GrimAA) | 1.1 | 0.23 | 1883.06 |
|  | 480698 (8.61% cases) | 0.0039 (GrimAA) | 1.15 | 0.44 | 1883.06 |
|  | 480698 (8.61% cases) | 0.0039 (GrimAA) | 1.2 | 0.67 | 1883.06 |
|  | **480698 (8.61% cases)** | **0.0039 (GrimAA)** | **1.25** | **0.85** | **1883.06** |
|  | 480698 (8.61% cases) | 0.0113 (HannumAA) | 1.1 | 0.54 | **5494.97** |
|  | **480698 (8.61% cases)** | **0.0113 (HannumAA)** | **1.15** | **0.87** | **5494.97** |
|  | **480698 (8.61% cases)** | **0.0113 (HannumAA)** | **1.2** | **0.98** | **5494.97** |
|  | **480698 (8.61% cases)** | **0.0113 (HannumAA)** | **1.25** | **1** | **5494.97** |
|  | 480698 (8.61% cases) | 0.0138 (PhenoAA) | 1.1 | 0.62 | 6727.46 |
|  | **480698 (8.61% cases)** | **0.0138 (PhenoAA)** | **1.15** | **0.92** | **6727.46** |
|  | **480698 (8.61% cases)** | **0.0138 (PhenoAA)** | **1.2** | **0.99** | **6727.46** |
|  | **480698 (8.61% cases)** | **0.0138 (PhenoAA)** | **1.25** | **1** | **6727.46** |
|  | **480698 (8.61% cases)** | **0.0272 (IEAA)** | **1.1** | **0.89** | **13441.6** |
|  | **480698 (8.61% cases)** | **0.0272 (IEAA)** | **1.15** | **1** | **13441.6** |
|  | **480698 (8.61% cases)** | **0.0272 (IEAA)** | **1.2** | **1** | **13441.6** |
|  | **480698 (8.61% cases)** | **0.0272 (IEAA)** | **1.25** | **1** | **13441.6** |

CKD, chronic kidney disease; eGFR, estimated glomerular filtration rate.

Notes:

1. The above estimates were based on Type-I error rate = 0.05.
2. True causal effect sizes were estimated based on double-standardized regression coefficients from the largest meta-analysis of EAA and kidney function led by Matías-García et al 2021 (beta ranges from -0.034 to -0.046 1 SD log-eGFR for 4 EAA). The unit of exposure and outcome were standardized for the power calculation (i.e. variance of the exposure variable and variance of the outcome variable are both equal to 1).
3. Levels of R^2^ were selected based on the variance explained by the genome-wide loci for each EAA from the EAA GWAS paper.
4. The SD of EAA ranges from 3.9 to 7.5 years, estimated based on the meta-analysis by Matías-García et al 2021. The SD of log(eGFR) is about 0.168, estimated based on an estimation from the UK Biobank data.
